# Privacy-aware adversarial defense with explainable AI for adversarial robustness in AI model

**DOI:** 10.1007/s40860-026-00273-7

**Published:** 2026-09-15

**Authors:** Bilal Sardar, Shareeful Islam, Stefano Silvestri, Spyridon Papastergiou

**Affiliations:** 1 School of Computing and Information Science, Anglia Ruskin University, East Road, Cambridge, UK; 2 Institute for High Performance Computing and Networking, National Research Council of Italy (ICAR-CNR), Via Pietro Castellino 111, 80131 Naples, Italy; 3 Research and Innovation, Maggioli S.p.A., Santarcangelo di Romagna, Italy; 4 Department of Informatics, University of Piraeus, Piraeus, Greece

**Keywords:** Differential privacy, Adversarial robustness, Unsupervised anomaly detection, Explainable AI, Latent space monitoring, Reliable intelligent environments, Fairness

## Abstract

AI models in critical sectors such as healthcare and finance must provide both data privacy and adversarial robustness. Differential Privacy (DP) protects training data by injecting noise, but this noise smooths decision boundaries and leaves models open to adversarial evasion, a tension known as the Privacy-Robustness Trade-off. Although this trade-off is well documented, its internal mechanism remains underexplored: prior work does not reveal how the noise reshapes a model’s reasoning or which features become vulnerable. To close this gap, we propose a Privacy-Aware Adversarial Defense grounded in Explainable AI. Specifically, we introduce the Attention Concentration Score (ACS), which measures how DP training shifts Transformer attention away from task-critical features toward non-functional ones. This attention drift correlates with adversarial vulnerability, providing a mechanistic explanation of the trade-off. Building on this insight, we develop a Manifold-Aligned Semantic Attack that targets the most drifted features, and a TrustScore defense that fuses embedding-level anomaly detection with attention-level consistency checks. We validate across two datasets (Adult Census, MIMIC-IV), two architectures (DeBERTa-V3-Large, LLaMA−3.1-8B), and seven experiments benchmarking five attacks against six defenses. Within the recommended range ($\epsilon \in [5, 10]$), models retain 84.2% accuracy (96.2% of baseline), while TrustScore reaches an Area Under the ROC Curve (AUC) of 0.87-−0.94, outperforming Isolation Forest (0.65) and supervised detection (0.58). Moreover, these conclusions hold under feature-categorization variants (attribution- and PCA-based); privacy noise disproportionately destabilizes the minority class; the consistency signal adds sub-millisecond overhead; and a surrogate-attention variant preserves detection under black-box deployment, establishing the approach’s dependability for reliable intelligent environments.

## Introduction

The growing deployment of Artificial Intelligence (AI) in sensitive domains such as healthcare, finance, and critical infrastructure has raised significant concerns about both data privacy and model security [1–3]. These concerns are especially acute in reliable intelligent environments, where AI models embedded in IoT-enabled clinical monitoring, smart-infrastructure analytics, and connected public-sector services operate continuously on personal and structured data and must remain simultaneously privacy-preserving and resilient to manipulation [4]. Structured tabular records—patient vitals, sensor and administrative metadata, demographic fields—constitute the dominant data modality in such environments, so the privacy–robustness behaviour of tabular classifiers is directly tied to the dependability of the environments that embed them. To address privacy risks, AI regulatory guidelines increasingly encourage the adoption of Differential Privacy (DP), particularly through Differentially Private Stochastic Gradient Descent (DP-SGD) training, which protects sensitive data by clipping per-example gradients and injecting calibrated noise [5]. However, this privacy-preserving noise simultaneously smooths the model’s decision boundaries, making it easier for adversarial inputs to cross those boundaries and evade detection [6]. This creates a tension known as the Privacy-Robustness Trade-off, where the mechanisms designed to protect data privacy can weaken the model’s resilience against adversarial attacks [7, 8].

Despite growing awareness of this trade-off, three key gaps remain in the current literature. First, existing work documents the tension between privacy and robustness but treats it as a black-box observation—without examining how privacy noise alters the model’s internal reasoning at the representation level. As a result, the mechanism by which DP degrades robustness remains unclear, limiting the design of targeted defenses. Second, current adversarial attacks against privacy-preserved models are privacy-agnostic: they use generic perturbation strategies such as Projected Gradient Descent (PGD) [9] and HopSkipJump [10] without exploiting the specific internal changes that DP introduces. This means existing threat assessments may underestimate the risk posed by adversaries who understand and target DP-induced weaknesses. Third, current detection methods rely on either supervised approaches trained on known attack patterns [11] or unsupervised monitors that assume adversarial inputs will deviate from the normal data distribution. Neither approach accounts for an attacker who deliberately crafts perturbations that remain statistically plausible, thereby evading standard detectors.

To address these interconnected gaps, this paper proposes a Privacy-Aware Adversarial Defense Approach that unifies mechanistic explanation, targeted attack design, and robust detection. Specifically, we investigate the following research questions:

- **RQ1 (Explainability):** How does DP-SGD training reshape the internal attention structure of AI models, and does the resulting attention drift correlate with increased adversarial vulnerability?
- **RQ2 (Attack):** Given that privacy noise shifts attention toward non-functional features, can an adversary exploit this drift to craft realistic perturbations that evade existing defenses?
- **RQ3 (Adversarial Defense):** Since such attacks remain within the data manifold, can combining embedding-level anomaly detection with attention-level consistency checks improve detection over either signal alone?
- **RQ4 (Privacy and Robustness Trade-off):** Across the full privacy spectrum, how do utility, attention drift, attack success, and detection performance co-evolve, and what operating range of ϵ jointly satisfies all four objectives?

In response to these questions, we make the following contributions:

- **Firstly,** we propose a Privacy-Aware Adversarial Defense approach organized into five sequential phases, with explainability as the central mechanism that drives both attack and defense design. Phase I prepares data with domain-informed feature categorization. Phase II trains models under DP-SGD across ten privacy budgets $\epsilon \in \{1, 2, 5, 8, 10, 15, 20, 30, 50, \infty \}$ using two architectures. Phase III introduces the Attention Concentration Score (ACS), an explainability metric that measures how privacy noise redistributes model attention from task-critical features toward non-functional attributes, and correlates this attention drift with adversarial vulnerability. This explainability insight then informs Phase IV, which constructs a manifold-aligned adversarial attack that targets the most drifted features, and Phase V, which builds a layered TrustScore defense combining embedding-level anomaly detection with attention-level consistency checks derived from the same explainability analysis.
- **Secondly,** we develop the technical components underlying each phase across three dimensions, all grounded in explainability. For explainability itself, we introduce the ACS metric validated against Integrated Gradients, and measure the drift-vulnerability relationship via Spearman correlation at the macro level and per-sample logistic regression at the micro level. For the attack, informed by the drift patterns discovered through explainability, we formalize two attacker variants under co-occurrence plausibility constraints: $\mathcal {A}_{\text {prac}}$ with uniform feature selection and $\mathcal {A}_{\text {info}}$ with drift-guided selection. For the defense, guided by the same attention analysis, we combine Isolation Forest on [CLS] embeddings with three attention consistency scoring variants (ratio-based $a_{\text {AC}}$, IG-based $a_{\text {AC}}^{\text {IG}}$, and JSD-based $a_{\text {JSD}}$), fused into a weighted TrustScore.
- **Thirdly,** to verify that these explainability-driven findings are not specific to a single model, we evaluate across two architectures: DeBERTa-V3-Large [12] with full DP-SGD and LLaMA−3.1-8B [13] with DP-LoRA (r=16). This cross-architecture validation tests whether the attention drift patterns, attack effectiveness, and defense performance generalize across different model scales and private training strategies.
- **Finally,** we validate the approach through seven experiments across two datasets—Adult Census Income [14] and MIMIC-IV [15]—that directly address the four research questions. Within the recommended privacy range ($\epsilon \in [5, 10]$), models retain up to 84.2% accuracy (96.2% of the non-private baseline), the drift-guided informed attacker consistently outperforms privacy-agnostic baselines, and the TrustScore-AC defense achieves AUC of 0.87-−0.94 across all attack types—outperforming both the Isolation Forest alone (AUC 0.65) and supervised detection (AUC 0.58) against manifold-aligned threats. Trade-off analysis across the full privacy spectrum reveals a non-linear co-evolution of utility, attention drift, attack success, and detection performance, with consistent trends preserved across both architectures. Beyond these core results, we establish the robustness and deployability of the approach through dedicated analyses of feature-categorization sensitivity (including attribution-derived and PCA-based partitions), class-conditional drift, an adaptive attention-mimicking adversary, surrogate-based detection for black-box APIs, inference-time overhead, and the fairness implications of privacy-induced attention shifts, demonstrating that the conclusions hold under realistic operating conditions for intelligent environments.

## Related work

This research is situated at the convergence of three interconnected domains: privacy-preserving model training, Transformer explainability, and adversarial attack and detection. While substantial progress has been made in each area individually, existing work has not simultaneously explained how privacy noise alters internal representations, constructed attacks that exploit this alteration, and designed defenses informed by the same analysis. The following subsections review relevant prior work and identify the specific gaps that motivate our approach.

### Privacy and adversarial robustness

Differential Privacy (DP) has become a widely adopted method for protecting training data, with DP-SGD [16] providing formal $(\epsilon , \delta )$-guarantees through per-example gradient clipping and calibrated noise injection, building on the theoretical foundations established by Dwork [5]. However, while DP protects sensitive data, it simultaneously introduces adversarial vulnerabilities. Specifically, Tursynbek et al. [6] demonstrated that DP-trained models can be *more* susceptible to adversarial perturbations than their non-private counterparts, and Boenisch et al. [8] further showed that unfavorable DP-SGD configurations can induce gradient masking, creating a false sense of security. Building on these observations, Zhang and Bu [17] investigated whether careful hyperparameter tuning can reconcile adversarial training with privacy guarantees, while Hayes et al. [18] provided theoretical bounds showing that the dimensionality-dependent noise in DP-SGD can widen the vulnerability surface. This dimensionality dependence is central to the mechanism we study: because the Gaussian noise injected by DP-SGD (Eq. 6) is isotropic and its calibrated standard deviation grows with the effective gradient dimension, high-dimensional models accumulate proportionally more perturbation along directions that are only weakly informative for the task. The net effect is a flattening of the decision surface in precisely those directions, so the margin an evasion attack must traverse to flip a prediction shrinks as privacy tightens. This provides the theoretical link between the dimensionality-dependent noise of DP-SGD and the ease with which a plausibility-constrained attack bypasses the smoothed boundary: the same noise that anonymizes the gradient also diffuses attention mass away from a small set of decisive features toward many low-information ones, and our Attention Concentration Score is designed to make this redistribution measurable. Although these studies collectively establish a clear privacy-robustness tension, they treat it as a black-box observation measured through external attack success rates, without examining *how* privacy noise reshapes the model’s internal representations. Our work directly addresses this gap through an attention drift analysis that provides the missing mechanistic explanation.

### Explainability in transformer-based models

Understanding the internal behaviour of Transformer models is essential for diagnosing why privacy noise degrades robustness. In this context, the use of attention weights as model explanations has been extensively debated. Jain and Wallace [19] showed that learned attention weights are frequently uncorrelated with gradient-based feature importance measures, while Wiegreffe and Pinter [20] countered that attention can still provide meaningful interpretability signals under appropriate diagnostic conditions. Rather than entering this debate, our work takes a *distributional* perspective: we measure how DP-SGD training systematically redistributes attention mass across feature categories and correlate these shifts with adversarial vulnerability. To guard against the limitations identified by Jain and Wallace, we validate all attention-based findings against Integrated Gradients [21], confirming that the observed redistribution reflects genuine representational shifts rather than attention-specific artifacts.Beyond attention, model-agnostic attribution methods such as SHAP [22] and LIME [23] offer architecture-independent ways to estimate feature importance; we use both, in addition to Integrated Gradients, to derive feature partitions automatically and to test whether our conclusions depend on a manually specified domain categorization (Sect. 4.9). Separately, the application of Transformers to structured tabular data has seen growing adoption. Hegselmann et al. [24] demonstrated that serializing tabular records into natural language enables large language models to perform competitively on classification tasks, while Dinh et al. [25] showed that such serialization supports few-shot learning on tabular benchmarks. These works establish the viability of Transformer-based tabular processing but do not examine how privacy-preserving training affects the resulting attention structure—a gap that our Phase III analysis addresses by quantifying attention drift in the context of serialized tabular inputs across ten privacy budgets.

### Adversarial attacks and detection

The privacy-robustness tension described above raises a natural follow-up question: can an informed adversary exploit the internal changes caused by DP to craft more effective attacks? Existing adversarial methods span a broad spectrum, from gradient-based approaches such as PGD [9] to decision-based attacks such as HopSkipJump [10], but these operate on continuous perturbation spaces and are not directly suited to the discrete features common in tabular data. For tabular domains, Ballet et al. [26] and Cartella et al. [27] proposed plausibility-constrained semantic perturbations that respect domain constraints such as feature interdependencies, yet neither incorporates knowledge of how privacy noise has shifted the model’s internal feature reliance. Our manifold-aligned attack (Phase IV) bridges this gap by combining statistical plausibility constraints with drift-informed feature selection, producing perturbations that are both semantically valid and targeted at the model’s privacy-induced weaknesses. Because a defense informed by explainability could itself be probed, we additionally consider adaptive adversaries in the sense of Athalye et al. [28] and Tramèr et al. [29], who argue that defenses must be evaluated against attackers aware of the detection mechanism; Sect. 4.10 reports such an evaluation.

On the detection side, unsupervised methods have shown promise for identifying adversarial inputs. The Isolation Forest algorithm [30] detects anomalies through random partitioning of latent feature space, while Fidel et al. [11] proposed using SHAP signatures computed on internal layers to discriminate between normal and adversarial inputs. Nevertheless, both approaches assume that adversarial inputs will deviate measurably from the authentic data distribution—an assumption that manifold-aligned attacks are specifically designed to violate. Our TrustScore defense (Phase V) addresses this limitation by fusing latent anomaly detection with attention consistency scoring, enabling robust detection against both off-manifold and manifold-aligned attacks that evade either signal alone.

In summary, existing work has established that DP degrades adversarial robustness [6, 8] and that attention mechanisms offer interpretability signals [19, 20], but to the best of our knowledge, these two observations have not been connected by explaining *how* privacy noise redistributes internal attention, constructing an attack that *exploits* this redistribution, and designing a defense that is *informed by* the same explainability analysis. This integrated perspective—linking privacy-induced representational shifts to both adversarial vulnerability and detection—is the central contribution of our work.

## Methodology: privacy-aware adversarial defense through explainability-driven analysis

**Fig. 1:**
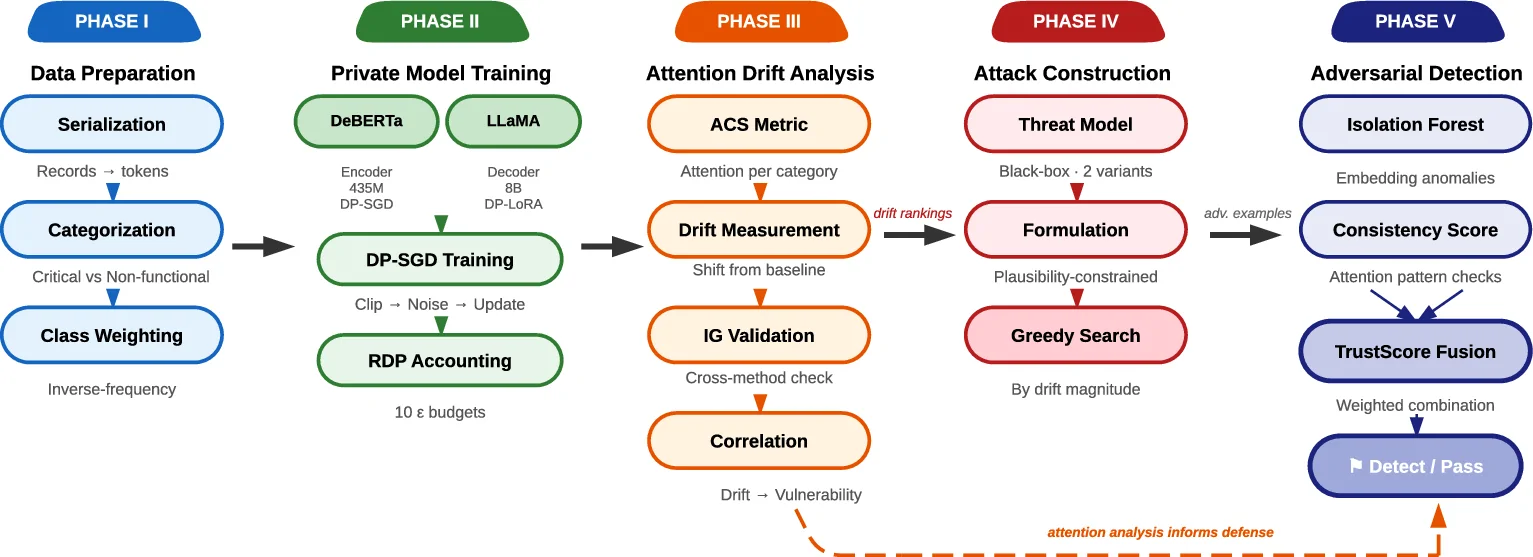
Privacy-Aware Adversarial Defense — Methodology Overview

This section presents the proposed Privacy-Aware Adversarial Defense. As illustrated in Fig. 1, it is organized into five sequential phases. Phase I (*Data Preparation*) serializes records and partitions features into task-critical and non-functional categories. Phase II (*Private Model Training*) trains DeBERTa-V3-Large (encoder, full DP-SGD) and LLaMA−3.1-8B (decoder, DP-LoRA) across ten privacy budgets. Phase III (*Attention Drift Analysis*) introduces the Attention Concentration Score, validates it against Integrated Gradients, and correlates drift with vulnerability. From Phase III the approach branches (dashed arrow in Fig. 1): drift rankings guide Phase IV (*Attack Construction*), a plausibility-constrained black-box semantic attack, while the same attention analysis supplies Phase V (*Adversarial Detection*) with the consistency score it fuses with an Isolation Forest into a TrustScore. We first fix notation, then describe each phase.

### Problem formulation and notation

Let $\mathcal {D} = \{(x_i, y_i)\}_{i=1}^{N}$ denote a training dataset where $x_i \in \mathcal {X}$ is a structured input record and $y_i \in \{0,1\}$ is the corresponding binary label. Let $f_\theta : \mathcal {X} \rightarrow [0,1]$ denote a Transformer-based classifier parameterized by θ, where the output represents the predicted probability of the positive class. We denote the [CLS] embedding produced by $f_\theta$ for input *x* as $\textbf{h}_\theta (x) \in \mathbb {R}^d$, where *d* is the hidden dimension of the Transformer. The attention weight assigned by head *k* from the [CLS] token to input token *j* at layer *l* is denoted $\alpha _{k,j}^{(l)}(x)$.

We consider models trained under $(\epsilon , \delta )$-differential privacy (DP), where the privacy budget ϵ>0 controls the strength of the guarantee—lower ϵ corresponds to stronger privacy—and δ>0 bounds the probability that the guarantee fails to hold. Our objective is threefold: (1) characterize how varying ϵ alters attention distributions and correlates with adversarial vulnerability, (2) construct an adaptive attack that exploits these privacy-induced shifts, and (3) develop a detection mechanism that remains effective against both standard and adaptive adversarial strategies. Achieving these objectives requires structured input representations that allow attention patterns to be meaningfully analyzed, which is the purpose of the first phase.

### Phase I: data preparation

Before training or analyzing any model, the raw structured data must be converted into a format where attention-based analysis is possible. This phase accomplishes two tasks: it serializes heterogeneous tabular records into tokenized sequences that the model can process, and it organizes the resulting tokens into meaningful feature categories that will be used to measure how privacy noise redistributes the model’s attention in Phase III.

#### Feature serialization and categorization

Feature serialization converts a structured record of mixed categorical, numerical, and textual fields into a single natural-language sequence. Each input record $x_i$ is transformed through a deterministic serialization function $T: \mathcal {X} \rightarrow \mathcal {S}$:

1 $$\begin{aligned} s_i = T(x_i) = \texttt {[CLS]}\ \oplus \ \bigoplus _{j=1}^{m} \left( \text {name}_j \texttt {:}\ \text {val}_j(x_i) \right) \ \oplus \ \texttt {[SEP]} \end{aligned}$$

where ⊕ denotes string concatenation, *m* is the total number of features, and $\text {name}_j$ and $\text {val}_j(x_i)$ are the name and value of the *j*-th attribute respectively. For example, a record with age 35 and occupation “Sales” is serialized as [CLS] age: 35 occupation: Sales [SEP]. This serialization enables the Transformer’s self-attention mechanism to capture cross-feature dependencies, rather than treating each feature as an isolated input.

Feature categorization partitions the serialized tokens into two disjoint groups based on domain knowledge, grounding the drift analysis in Phase III:

2 $$\begin{aligned} \mathcal {T}(s_i) = \mathcal {T}_{\text {critical}}(s_i) \cup \mathcal {T}_{\text {non-func}}(s_i), \quad \mathcal {T}_{\text {critical}} \cap \mathcal {T}_{\text {non-func}} = \emptyset \end{aligned}$$

Here, $\mathcal {T}_{\text {critical}}$ contains tokens corresponding to task-relevant features whose values are directly informative for the classification task (e.g., diagnostic codes, risk indicators), while $\mathcal {T}_{\text {non-func}}$ contains tokens corresponding to non-functional attributes that are not inherently predictive but may be exploited by the model under noisy training conditions (e.g., administrative metadata, demographic identifiers). Structural tokens ([CLS], [SEP], and template words such as feature names) are excluded from both sets, as they do not carry record-specific information.

Because this partition is the entry point of the entire approach, it is important to be explicit about how it is obtained and how sensitive the downstream conclusions are to it. Three points clarify its role. First, the categorization need not be a binary statement about whether a feature *causes* the outcome; it is an operational grouping into features the deployer treats as decision-relevant versus auxiliary, and features whose status is genuinely ambiguous (for example, age in Adult Census, which is plausibly predictive yet also a demographic attribute) can be assigned to either group, or duplicated into a separate “ambiguous” bucket, without changing the ACS definition, which simply sums attention mass over whichever token set is supplied. Second, when no reliable domain prior exists—or when features are derived through transformations such as Principal Component Analysis (PCA) that lack human-interpretable names—the partition can be produced automatically from an attribution method (SHAP [22], LIME [23], or Integrated Gradients [21]) computed on the *non-private* reference model: features whose attribution exceeds the median are assigned to $\mathcal {T}_{\text {critical}}$ and the remainder to $\mathcal {T}_{\text {non-func}}$. For PCA-derived inputs, attribution is computed in the component space and the resulting component groups play the role of feature categories. Third, the central claim—that private training shifts attention *away from* the high-importance group—is by construction a *relative* comparison between the private and non-private model under the *same* partition; any systematic mislabelling therefore biases $\text {ACS}_{\text {critical}}(\infty )$ and $\text {ACS}_{\text {critical}}(\epsilon )$ in the same direction and largely cancels in the drift $\Delta _{\text {critical}}(\epsilon )$. We verify all three points empirically in Sect. 4.9, where we re-run the full analysis under swapped, attribution-derived, and PCA-based partitions and confirm that the drift–vulnerability correlation is preserved.

#### Class imbalance handling

When one class is underrepresented the model may learn to ignore it, an effect amplified by private-training noise. Data-level augmentation techniques such as SMOTE [31] are generally discouraged in private training when generated from private records, because synthetic samples produced via nearest-neighbor interpolation may encode information about individual training instances and oversampling changes the data distribution seen by the training algorithm, complicating the privacy interpretation and the accounting assumptions underlying privacy amplification by subsampling [32]. We therefore adopt *inverse-frequency class weighting* applied directly to the loss function:

3 $$\begin{aligned} w_c = \frac{N}{|\mathcal {C}| \cdot N_c} \end{aligned}$$

where $N_c$ is the number of training samples in class *c* and $|\mathcal {C}|$ is the number of classes. The weighted loss for a sample $(s_i, y_i)$ is $\mathcal {L}_w = w_{y_i} \cdot \mathcal {L}(f_\theta (s_i), y_i)$. This reweighting modifies gradient magnitudes without altering the dataset or the privacy accounting, because the weight $w_c$ is a deterministic function of public class counts and does not depend on any individual record’s content. Data loaders are configured with Poisson sampling at rate q=B/N, where *B* is the batch size, satisfying the subsampling requirement for privacy amplification [16, 32]. We note, however, that inverse-frequency weighting equalizes the *expected* loss contribution of each class but not the *variance* injected by per-sample clipping and Gaussian noise: minority-class gradients appear in fewer sampled batches, so their clipped contributions are perturbed by proportionally more noise per effective update. This asymmetry leads us to expect—and Sect. 4.4.1 confirms—that attention drift and the resulting vulnerability are concentrated in the minority class.

The output of Phase I is a set of serialized sequences $\{s_i\}_{i=1}^N$ with associated feature categorizations and class weights, ready for private training in Phase II.

### Phase II: private model training

The second phase builds the classification models and trains them across privacy budgets, producing the controlled conditions needed to isolate the effect of privacy noise. To ensure that our findings are not artifacts of a single architecture, we employ two structurally distinct Transformer models: DeBERTa-V3-Large [12], a high-performing encoder model that serves as the primary backbone and is trained with full DP-SGD, and LLaMA−3.1-8B [13], a large-scale decoder model that serves as the secondary backbone and is trained with parameter-efficient DP-LoRA [33]. This pairing tests whether the observed phenomena generalize across structurally different attention mechanisms (bidirectional vs. causal), model scales (435 M vs. 8B parameters), and private training strategies (full fine-tuning vs. adapter-based).

#### Building the classification models

We employ two models in a primary–secondary configuration: DeBERTa-V3-Large, an encoder under full private fine-tuning that is the main subject of all analyses, and LLaMA−3.1-8B, a decoder under parameter-efficient private adaptation that validates cross-architecture generalization.

***Primary model: DeBERTa-V3-Large***

We adopt DeBERTa-V3-Large [12] as the primary Transformer backbone. DeBERTa-V3-Large is a 24-layer, 16-head, 1024-dimensional encoder Transformer (∼435 M parameters) that introduces *disentangled attention*, which separates content and position representations into distinct vectors and computes attention using both. We select this model for three reasons: (i) it achieves strong performance among encoder models on SuperGLUE and a wide range of NLP benchmarks, establishing a strong non-private baseline; (ii) its disentangled attention mechanism provides a richer decomposition of attention patterns for drift analysis, since content-based and position-based attention can be examined separately; and (iii) its moderate size permits full DP-SGD fine-tuning with per-sample gradient computation via microbatching, feasible on a single A100 40GB GPU. For classification, we append a linear projection head on top of the [CLS] token representation:

4 $$\begin{aligned} f_\theta (s) = \sigma \left( \textbf{w}^\top \textbf{h}_\theta ^{(L)}(s) + b\right) \end{aligned}$$

where $\textbf{h}_\theta ^{(L)}(s) \in \mathbb {R}^{1024}$ is the [CLS] embedding from the final layer L=24, $\textbf{w} \in \mathbb {R}^{1024}$ and $b \in \mathbb {R}$ are learnable parameters, and σ denotes the sigmoid activation function.

***Secondary model: LLaMA−3.1-8B***

To evaluate whether the observed attention drift generalizes to decoder-only architectures, we additionally train LLaMA−3.1-8B [13], a 32-layer, 32-head, 4096-dimensional causal Transformer (∼8B parameters). LLaMA differs from DeBERTa in fundamental ways: it uses causal (unidirectional) attention rather than bidirectional, Grouped Query Attention (GQA) with 8 key-value heads for efficiency, and RoPE positional embeddings rather than disentangled position encoding. Since full DP-SGD is computationally prohibitive at 8B parameters (per-sample gradient clipping requires storing individual gradients for every sample), we apply DP-LoRA [33], which restricts trainable parameters to low-rank adapter matrices (r=16) inserted into the query and value projections. The privacy guarantee is applied to these adapter parameters only, while the frozen backbone does not require privacy accounting. For classification, we use the hidden state of the final token (analogous to [CLS] in encoder models) with a linear head. Because the backbone remains frozen, DP-LoRA exposes the model to privacy noise only along the low-rank adapter directions, which is precisely why we treat LLaMA as a secondary, complementary configuration rather than a like-for-like replica of full DP-SGD: it isolates the effect of restricting noise to a small adapter subspace, and the fact that the pre-trained backbone is intact means its drift magnitudes are an attenuated lower bound on what a decoder trained privately from scratch would exhibit. We return to this interpretation in the discussion. Observing consistent attention drift patterns across both an encoder with disentangled bidirectional attention (DeBERTa, 435 M, full DP-SGD) and a decoder with causal attention (LLaMA, 8B, DP-LoRA) would provide strong evidence that the phenomenon is fundamental to the interaction between privacy noise and Transformer attention, rather than an artifact of a specific architecture, scale, or training strategy.

#### Private training procedure

Private training modifies the standard gradient descent procedure to ensure that the learned model does not reveal whether any particular record was included in the training data. Given the serialized training data from Phase I, we fine-tune each model using DP-SGD [16]. Standard SGD computes the parameter update $\theta _{t+1} = \theta _t - \eta \cdot \frac{1}{|\mathcal {B}_t|} \sum _{i \in \mathcal {B}_t} \nabla _\theta \mathcal {L}_w(f_\theta (s_i), y_i)$ for a mini-batch $\mathcal {B}_t$ at step *t*. DP-SGD modifies this through two operations that together bound the influence of any single training instance on the learned parameters.

First, each per-sample gradient $\textbf{g}_i = \nabla _\theta \mathcal {L}_w(f_\theta (s_i), y_i)$ is clipped to bound its $\ell _2$ norm, ensuring that no single record contributes more than *C* to the aggregate gradient:

5 $$\begin{aligned} \bar{\textbf{g}}_i = \textbf{g}_i \cdot \min \left( 1, \frac{C}{\Vert \textbf{g}_i\Vert _2}\right) \end{aligned}$$

where C>0 is the clipping threshold, a hyperparameter that controls the sensitivity of the training procedure. Second, calibrated Gaussian noise is added to the aggregated clipped gradients:

6 $$\begin{aligned} \tilde{\textbf{g}}_t = \frac{1}{|\mathcal {B}_t|} \left( \sum _{i \in \mathcal {B}_t} \bar{\textbf{g}}_i + \mathcal {N}(\textbf{0}, \sigma ^2 C^2 \textbf{I}) \right) \end{aligned}$$

where σ is the noise multiplier that determines the magnitude of the injected noise, and $\textbf{I}$ is the identity matrix. The noisy gradient $\tilde{\textbf{g}}_t$ is then used for the parameter update $\theta _{t+1} = \theta _t - \eta \cdot \tilde{\textbf{g}}_t$. The combination of gradient clipping and noise injection allows the training algorithm to satisfy $(\epsilon , \delta )$-differential privacy, meaning that the learned model $f_{\theta _\epsilon }$ does not reveal whether any particular record was included in the training set, up to the privacy parameters ϵ and δ.

#### Privacy accounting and budget spectrum

Privacy accounting tracks the privacy consumed during training so the total stays within the target budget. We use the Rényi Differential Privacy (RDP) accountant [34], which provides tighter composition bounds than the original moments accountant. A single step of the subsampled Gaussian mechanism with sampling rate q=B/N and noise multiplier σ satisfies $(\alpha , \varrho (\alpha ))$-RDP. The standard upper bound for integer orders $\alpha \ge 2$ is:

7 $$\begin{aligned} \varrho (\alpha ) \le \frac{1}{\alpha - 1} \log \left( \sum _{k=0}^{\alpha } \left( {\begin{array}{c}\alpha \\ k\end{array}}\right) q^k (1-q)^{\alpha -k} e^{k(k-1)/(2\sigma ^2)} \right) \end{aligned}$$

We use this standard upper bound as implemented in Opacus [35]. Over *T* training steps, the RDP guarantee composes linearly: $\varrho _T(\alpha ) = T \cdot \varrho (\alpha )$. The final $(\epsilon , \delta )$-DP guarantee is then obtained by converting the RDP bound:

8 $$\begin{aligned} \epsilon = \min _{\alpha > 1} \left( \varrho _T(\alpha ) + \frac{\log (1/\delta )}{\alpha - 1} \right) \end{aligned}$$

For each target budget $\epsilon _{\text {target}}$, we solve for the noise multiplier σ that satisfies Eq. 8 given fixed *T*, *q*, and $\delta = 10^{-5}$. We set $\delta = 10^{-5}$ as a default consistent with common practice; for datasets where $1/N < 10^{-5}$ (i.e., $N > 10^5$), we additionally report results with δ=1/N as a sensitivity check. We train separate model instances at $\epsilon \in \{1, 2, 5, 8, 10, 15, 20, 30, 50, \infty \}$, where $\epsilon = \infty$ denotes the non-private baseline (standard fine-tuning without clipping or noise). This range of ten configurations spans from strong privacy (ϵ=1) through moderate regimes commonly used in practice [36] (ϵ=10) to relaxed settings (ϵ=50), providing sufficient data points for the correlation analysis in Phase III. We report the exact $(\epsilon , \delta )$ achieved after training for each model.

Phase II outputs a spectrum of models $\{f_{\theta _\epsilon }\}_{\epsilon }$ for both architectures, each evaluated on $\mathcal {D}_{\text {val}}$ for baseline accuracy and $F_1$.

### Phase III: attention drift analysis

The third phase analyzes how private training reshapes the internal attention structure, establishing a quantitative link between the privacy budget and the model’s feature attribution that drives both the attack in Phase IV and the defense in Phase V.

#### Justification for attention-based explainability

Using attention weights as explanations has been debated in the interpretability literature. Jain and Wallace [19] showed that attention weights do not always correlate with gradient-based feature importance, while Wiegreffe and Pinter [20] demonstrated that attention weights *can* provide meaningful explanations when the architecture constrains information flow through the attended representations. In our setting, three factors support the use of attention as the primary explainability signal. First, in both architectures, the classification decision depends on a single readout token—[CLS] for DeBERTa, the final token for LLaMA—and the attention weights from this readout token to all input tokens directly determine which features influence the prediction (Eq. 4), satisfying the information-flow condition identified by Wiegreffe and Pinter [20]. Second, we use attention as a *relative* diagnostic—comparing the same metric across private and non-private models trained on identical data—rather than claiming absolute feature attributions. This comparative use is less susceptible to the concerns of Jain and Wallace [19], as systematic biases would affect both models equally and cancel out in the drift computation. Third, we empirically validate against Integrated Gradients (IG) [21], an axiomatic gradient-based attribution method, by defining an IG-based concentration score:

9 $$\begin{aligned} \text {IGS}_c(\epsilon ) = \frac{1}{|\mathcal {D}_{\text {eval}}|} \sum _{s \in \mathcal {D}_{\text {eval}}} \sum _{j \in \mathcal {T}_c(s)} \left| \text {IG}_j(s; f_{\theta _\epsilon }) \right| \end{aligned}$$

where $\text {IG}_j(s; f_{\theta _\epsilon })$ is the integrated gradient attribution for token *j*, computed by accumulating gradients along a straight-line path from a fixed baseline embedding to the actual input embedding. The corresponding IG-based drift is $\Delta _c^{\text {IG}}(\epsilon ) = \text {IGS}_c(\infty ) - \text {IGS}_c(\epsilon )$. A strong Spearman rank correlation between $\Delta _c(\epsilon )$ and $\Delta _c^{\text {IG}}(\epsilon )$ across the privacy spectrum would confirm that the attention-based drift captures a genuine representational shift rather than an attention-specific artifact.

#### Attention concentration score and drift

The Attention Concentration Score (ACS) quantifies how much of a model’s total attention mass is directed toward a feature category, summarizing its focus on task-critical versus non-functional attributes. For a given input *s* and trained model $f_{\theta _\epsilon }$, the multi-head self-attention mechanism at layer *l* produces attention weights $\alpha _{k,j}^{(l)}(s)$ for each head $k \in \{1, \ldots , H\}$ and token position *j*. For encoder models (DeBERTa), we analyze the [CLS]-to-token attention from the final layer, where each weight represents the contribution of token *j* to the classification readout. For decoder models (LLaMA), we analyze the final-token-to-context attention, where the final token serves as the classification readout under the causal attention mask. In both cases, we extract attention from the final layer *L*, as it most directly governs the classification decision. The ACS for a feature category $c \in \{\text {critical}, \text {non-func}\}$ under privacy budget ϵ is:

10 $$\begin{aligned} \text {ACS}_c(\epsilon ) = \frac{1}{|\mathcal {D}_{\text {eval}}|} \sum _{s \in \mathcal {D}_{\text {eval}}} \frac{1}{H} \sum _{k=1}^{H} \sum _{j \in \mathcal {T}_c(s)} \alpha _{k,j}^{(L)}(s) \end{aligned}$$

where $\mathcal {T}_c(s)$ is the set of token positions belonging to category *c* in the serialized input *s*. A higher $\text {ACS}_{\text {critical}}$ indicates that the model concentrates more attention on task-relevant features.

Attention drift measures the change in this concentration under private training relative to the non-private baseline:

11 $$\begin{aligned} \Delta _c(\epsilon ) = \text {ACS}_c(\infty ) - \text {ACS}_c(\epsilon ) \end{aligned}$$

A positive $\Delta _{\text {critical}}(\epsilon )$ indicates that the private model allocates less attention to task-critical features than the non-private baseline, while a negative $\Delta _{\text {non-func}}(\epsilon )$ indicates increased attention to non-functional attributes. We hypothesize that $\Delta _{\text {critical}}(\epsilon )$ increases monotonically as ϵ decreases (stricter privacy), and that this drift correlates with adversarial vulnerability.

In addition to category-level aggregation, we define a *per-feature* ACS that measures the attention mass directed toward an individual feature *j*, aggregated across all tokens belonging to that feature:

12 $$\begin{aligned} \text {ACS}_j(\epsilon ) = \frac{1}{|\mathcal {D}_{\text {eval}}|} \sum _{s \in \mathcal {D}_{\text {eval}}} \frac{1}{H} \sum _{k=1}^{H} \sum _{t \in \text {tokens}(j,s)} \alpha _{k,t}^{(L)}(s) \end{aligned}$$

where $\text {tokens}(j, s)$ denotes the set of token positions corresponding to feature *j* in the serialized input *s*. The per-feature drift $\Delta _j(\epsilon ) = \text {ACS}_j(\infty ) - \text {ACS}_j(\epsilon )$ quantifies how much attention a specific feature gains or loses under private training, and is used in Phase IV to rank features for adversarial targeting (Eq. 16).

Because non-functional and task-critical features are sometimes statistically correlated (for example, in Adult Census relationship and marital-status are correlated with the income label even though we treat the former as non-functional), a natural question is whether a feature labelled non-functional that carries indirect predictive signal would be misread by the drift analysis. The per-feature ACS resolves this cleanly: $\text {ACS}_j$ measures the attention the readout token actually allocates to feature *j*, irrespective of *j*’s category label, so an indirectly informative non-functional feature simply registers as one that *gains* attention under private training—which is exactly the behaviour the attack in Phase IV is designed to exploit. The category label affects only how we *aggregate* per-feature drift into the macro-level summary $\Delta _{\text {critical}}$; it never affects whether an individual feature’s drift is measured correctly. We confirm in Sect. 4.9 that moving such correlated features between categories leaves the per-feature drift rankings, and therefore the attack targets, essentially unchanged.

#### Drift-vulnerability correlation

We test whether the attention redistribution translates into increased adversarial susceptibility at two levels of granularity. At the *macro level*, we compute the Spearman rank correlation $\rho _s$ between $\Delta _{\text {critical}}(\epsilon )$ and the semantic attack success rate $\text {ASR}(\epsilon )$ across the ten privacy budgets. We use Spearman rather than Pearson because the hypothesis is monotonic but not necessarily linear, and Spearman is more robust with a moderate number of points. We report 95% bootstrap confidence intervals (1000 resamples) for $\rho _s$. At the *micro level*, we compute per-sample drift-vulnerability correlation: for each test sample *s* and each ϵ, we measure the sample-level attention drift $\Delta _{\text {critical}}^{(s)}(\epsilon ) = \text {ACS}_{\text {critical}}^{(s)}(\infty ) - \text {ACS}_{\text {critical}}^{(s)}(\epsilon )$ and the binary attack outcome (success/failure), then fit a logistic regression of attack success on sample-level drift, reporting the odds ratio and its significance. This per-sample analysis provides thousands of observations rather than relying on ten aggregate values.

Phase III outputs per-feature and per-category drift measurements with correlation statistics linking drift to vulnerability, which inform Phase IV and Phase V.

### Phase IV: attack construction

The fourth phase designs an adversarial attack that exploits the attention drift from Phase III, formalizing an attacker who targets the most vulnerable features while remaining indistinguishable from authentic data. We operationalize “manifold-aligned” as satisfying the co-occurrence plausibility constraint defined below; we empirically validate this alignment via embedding-distance distributions and Isolation Forest anomaly scores in Sect. 4.

#### Threat model

We define the adversary’s knowledge, capabilities, and constraints as follows:

- *Knowledge:* The adversary knows the input feature schema (feature names, domains, and value types) and has access to a publicly available co-occurrence distribution $\mathcal {P}$ over feature values, estimated from census or domain-specific public data. The adversary does *not* have access to the model’s private weights $\theta _\epsilon$, gradients, or the private training data $\mathcal {D}$.
- *Query access:* The adversary can submit inputs to the deployed model and observe the predicted label $\hat{y} = \mathbb {1}[f_{\theta _\epsilon }(T(x)) > 0.5]$ and the prediction confidence $f_{\theta _\epsilon }(T(x))$. This corresponds to a *score-based black-box* threat model, realistic for deployed ML-as-a-Service APIs [37].
- *Feature selection (two variants):* We evaluate two attacker variants. The **practical attacker** ($\mathcal {A}_{\text {prac}}$) selects mutable features uniformly from $\mathcal {T}_{\text {non-func}}$, using only public feature priors and query feedback. The **informed attacker** ($\mathcal {A}_{\text {info}}$) selects features based on attention drift rankings from Phase III (Eq. 16), obtainable via an explanation interface or surrogate estimation. Comparing the two isolates the security risk of interpretability exposure.
- *Objective:* Flip the model’s prediction ($\hat{y} \ne y$) while ensuring the perturbed input remains statistically consistent with the authentic data distribution.
- *Budget:* The adversary is limited to $Q_{\max }$ queries per attack attempt. The theoretical maximum is $Q_{\max } = \sum _{j \in \mathcal {F}_{\text {mut}}} |\mathcal {V}_j^{\text {valid}}|$, the total number of plausible substitutions across all mutable features; in practice, we impose a fixed cap of $Q_{\max } = 500$ to reflect realistic API rate limits (Sect. 4).

This threat model is weaker than the white-box assumption used by PGD [9] and more informative than the decision-only model used by HopSkipJump [10], positioning our attack in a practically relevant middle ground. The assumption of a publicly available co-occurrence distribution $\mathcal {P}$ deserves scrutiny, because in highly proprietary medical or industrial settings the attacker may not have access to a statistically faithful $\mathcal {P}$. We treat the perfectly-known $\mathcal {P}$ as a conservative, worst-case threat that upper-bounds the practical attacker rather than as a typical condition. To quantify how the assumption affects realism, Sect. 4.10 re-runs $\mathcal {A}_{\text {info}}$ with a degraded co-occurrence model estimated from only a 10% public subsample and from a distributionally shifted public corpus, and reports the corresponding reduction in attack success; the gap between this realistic setting and the idealized one bounds the extent to which the headline numbers overstate the practical risk.

#### Attack formulation

The adversary optimizes an objective that balances flipping the prediction against maintaining statistical plausibility. Let $$\mathcal {P}$$ denote the publicly available co-occurrence distribution. For a perturbed record $x^{\prime }$ obtained by modifying features in $$\mathcal {F}_{\text {mut}} \subseteq \{1, \ldots , m\}$$, we define the *statistical plausibility score* as the product of conditional probabilities:

13 $$\begin{aligned} S(x') = \prod _{j \in \mathcal {F}_{\text {mut}}} P_{\mathcal {P}}\left( x'_j \mid x'_{\setminus j}\right) \end{aligned}$$

where $P_{\mathcal {P}}(x'_j \mid x'_{\setminus j})$ is the conditional probability of the perturbed value $x'_j$ given all other feature values, estimated from $\mathcal {P}$. This score measures how natural the perturbed record appears relative to authentic data. For categorical features the conditional is a normalized contingency-table count smoothed with a Dirichlet prior. For *continuous* features, where the contingency table is undefined, we estimate $P_{\mathcal {P}}(x'_j \mid x'_{\setminus j})$ with a conditional Gaussian kernel-density estimate fitted on the public data, evaluated at $x'_j$ and conditioned on the nearest-neighbour bin of $x'_{\setminus j}$; equivalently, the attacker may discretize each continuous feature into deciles and reuse the categorical estimator. Both estimators are computed once, offline, from public statistics and add no query cost. The plausibility threshold $\tau _p$ is not hand-tuned but *calibrated* as the first-percentile plausibility score of authentic held-out records, so that by construction at least 99% of genuine records satisfy $S(x)\ge \tau _p$; this is the source of the value $\tau _p=0.01$ used in Sect. 4, and Sect. 4.10 reports the attack’s sensitivity to this choice. Because the threshold is a percentile of an empirical score distribution rather than an absolute probability, it is invariant to feature cardinality: for high-cardinality categorical features—where individual conditional probabilities are small—the Dirichlet smoothing keeps the estimates well-defined and the percentile calibration automatically rescales $\tau _p$ to the observed distribution, so no per-feature retuning is required. The attack then solves the following constrained optimization:

14 $$\begin{aligned}  &   x^* = \arg \max _{x' \in \mathcal {X}} f_{\theta _\epsilon }(T(x'))_{[\lnot y]} \quad \text {s.t.} \quad S(x') \ge \tau _p, \nonumber \\  &   \quad x'_j = x_j \ \forall j \notin \mathcal {F}_{\text {mut}} \end{aligned}$$

where $f_{\theta _\epsilon }(T(x'))_{[\lnot y]}$ denotes the model’s confidence in the incorrect class, defined for binary classification as:

15 $$\begin{aligned} f_{\theta _\epsilon }(T(x'))_{[\lnot y]} = {\left\{ \begin{array}{ll} f_{\theta _\epsilon }(T(x')) &  \text {if } y = 0 \\ 1 - f_{\theta _\epsilon }(T(x')) &  \text {if } y = 1 \end{array}\right. } \end{aligned}$$

This quantity is directly observable via query access. The plausibility threshold $\tau _p$ ensures the perturbation remains within the authentic data distribution, and the mutable feature set $\mathcal {F}_{\text {mut}}$ is determined by the feature selection strategy described next.

#### Feature selection and search

For the informed attacker $\mathcal {A}_{\text {info}}$, the set of mutable features is selected based on the attention drift from Phase III:

16 $$\begin{aligned} \mathcal {F}_{\text {mut}} = \left\{ j \in \mathcal {T}_{\text {non-func}} : |\Delta _j(\epsilon )| > \tau _d \right\} \end{aligned}$$

where $\Delta _j(\epsilon ) = \text {ACS}_j(\infty ) - \text {ACS}_j(\epsilon )$ is the per-feature attention drift defined in Eq. 12 and $\tau _d$ is the drift threshold. Features that private training has made the model disproportionately sensitive to are precisely those the attacker targets. For the practical attacker $\mathcal {A}_{\text {prac}}$, $\mathcal {F}_{\text {mut}}$ is drawn uniformly from $\mathcal {T}_{\text {non-func}}$.

**Table 1:** Asymptotic cost of the five attacks

Attack	Query model	Cost	Dim. dependent?
PGD	WB	$\mathcal {O}(I\,|\theta |)$	Yes
HopSkipJump	DEC	$\mathcal {O}(I\,d)$	Yes
Random semantic	SCORE	$\mathcal {O}(FV)$	No
$\mathcal {A}_{\text {prac}}$	SCORE	$\mathcal {O}(FV)\!\le \! Q_{\max }$	No
$\mathcal {A}_{\text {info}}$	SCORE	$\mathcal {O}(F\log F + FV)\!\le \! Q_{\max }$	No

*I*: iterations; |θ|: parameter count; *d*: input dimension; *F*: mutable features; *V*: plausible substitutions per feature. Query model: WB = white-box gradients, DEC = decision-only, SCORE = score-based

Given the discrete nature of the feature space and the score-based query access, we solve Eq. 14 via greedy combinatorial search (Algorithm 1). The algorithm iterates over features in descending order of $|\Delta _j(\epsilon )|$, filters candidate substitutions by plausibility, queries the model for each candidate, and retains the substitution that maximizes confidence in the incorrect class while satisfying the plausibility constraint. A query counter enforces the budget $Q_{\max }$.

**Algorithm 1:**
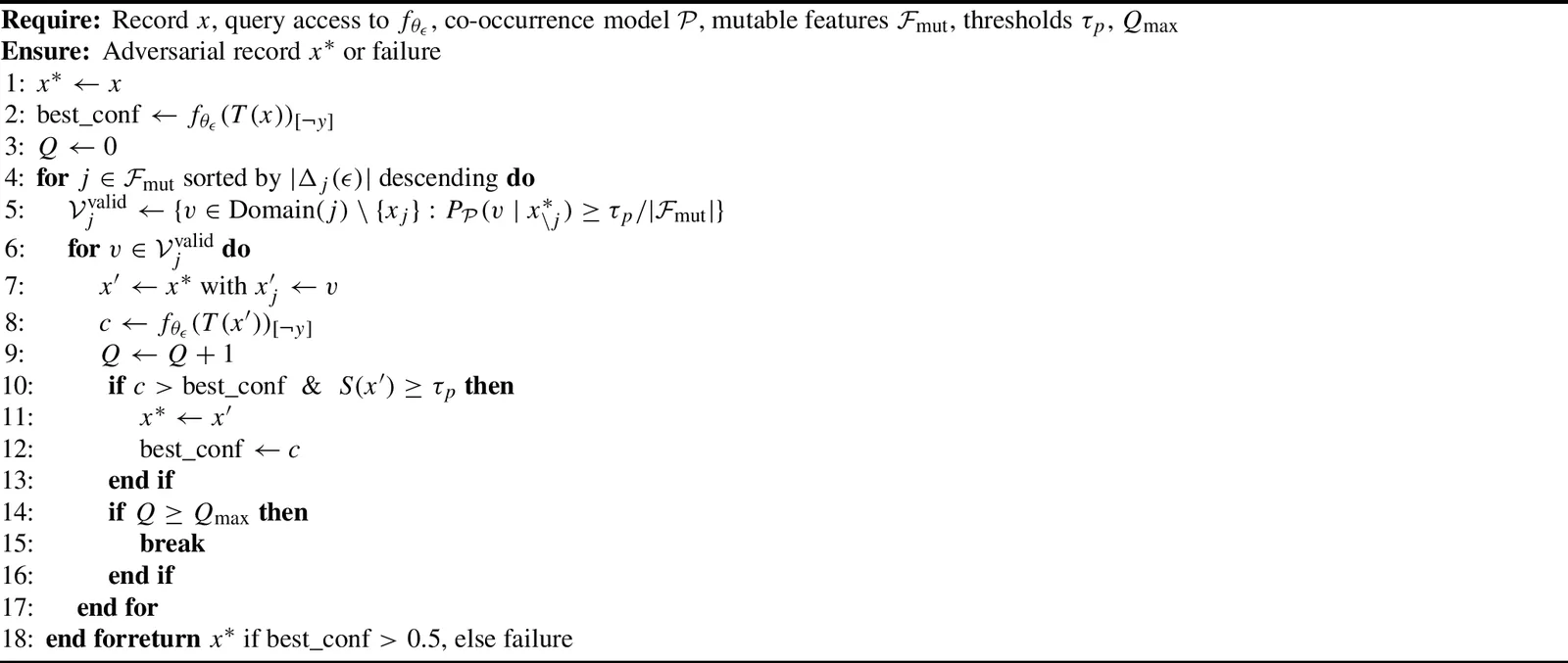
Manifold-Aligned Semantic Attack

***Complexity and convergence***

Because the reviewers ask how the cost of this attack compares with continuous baselines, we state its complexity explicitly. Let $F=|\mathcal {F}_{\text {mut}}|$ be the number of mutable features and $V=\max _j |\mathcal {V}_j^{\text {valid}}|$ the largest plausible substitution set after filtering. The greedy search performs at most F·V model queries, so its query complexity is $\mathcal {O}(FV)$, hard-capped at $Q_{\max }$; the offline plausibility filtering is $\mathcal {O}(FV)$ table look-ups with no query cost, and sorting features by drift is $\mathcal {O}(F\log F)$. The attack is therefore polynomial in the number of features and *independent of model dimension*, in contrast to PGD, whose cost $\mathcal {O}(I\,|\theta |)$ scales with the iteration count *I* and the full parameter count |θ| and additionally requires white-box gradients, and HopSkipJump, whose decision-based gradient estimation needs $\mathcal {O}(I\,d)$ queries with large constants. Table 1 summarizes this comparison. The drift ordering does more than bound the worst case: because the most-drifted features are tried first, $\mathcal {A}_{\text {info}}$ reaches a successful flip after far fewer queries than the budget allows—on average 182 queries on Adult and 241 on MIMIC-IV, versus 356 and 431 for the uniformly-ordered $\mathcal {A}_{\text {prac}}$—so the informed ordering improves both the success rate and the convergence rate while operating strictly within the same $Q_{\max }=500$ ceiling.

#### Adaptive adversary

The strongest threat to a defense that relies on attention consistency is an adversary who is aware of the detector and optimizes the attack to evade it. We therefore define an *adaptive* variant of $\mathcal {A}_{\text {info}}$ that, in addition to flipping the prediction, constrains its perturbations to preserve the authentic critical-to-non-functional attention ratio. Concretely, it augments the objective of Eq. 14 with a penalty $|R(x')-\mu _R|$, where R(·) and $\mu _R$ are the consistency ratio and its clean-data mean defined in Phase V (Eqs. 18–19), accepting a substitution only if it both raises misclassification confidence and keeps $|R(x')-\mu _R|$ below the detection threshold. This requires the attacker to estimate R(·), hence access to attention through an exposed explanation interface or the surrogate estimator of Phase V. Section 4.10 evaluates this attacker.

Phase IV outputs adversarial examples for each variant and budget, used as test inputs for Phase V.

### Phase V: adversarial detection

The fifth phase builds a layered detector that combines two complementary signals from the model’s internal representations: latent anomaly detection on the embedding space and attention consistency scoring on the attention patterns, fused into a single trust score that flags or passes each input.

#### Latent anomaly detection

Latent anomaly detection flags inputs whose Transformer embeddings fall outside the distribution of authentic data. We train an Isolation Forest [30] on the [CLS] embeddings of authentic (non-adversarial) evaluation data:

17 $$\begin{aligned} \mathcal {M}_{\text {IF}} = \text {IsolationForest}\left( \left\{ \textbf{h}_{\theta _\epsilon }(s) : s \in \mathcal {D}_{\text {auth}} \right\} \right) \end{aligned}$$

At inference, the Isolation Forest assigns an anomaly score $a_{\text {IF}}(x) \in [-1, 1]$ to each input based on the average path length required to isolate its embedding in randomly constructed trees. Inputs with highly negative scores deviate from the authentic embedding distribution and are considered anomalous. This component is effective against attacks that push representations away from the learned data manifold (e.g., PGD, DeepFool), but may miss manifold-aligned perturbations that preserve embedding proximity.

#### Attention consistency scoring

Attention consistency scoring catches inputs that evade embedding-level detection by checking whether the model’s attention pattern is consistent with authentic data. The primary score computes the ratio of attention allocated to task-critical versus non-functional features for a given input *s*:

18 $$\begin{aligned} R(s) = \frac{\frac{1}{H}\sum _{k=1}^{H} \sum _{j \in \mathcal {T}_{\text {critical}}(s)} \alpha _{k,j}^{(L)}(s)}{\frac{1}{H}\sum _{k=1}^{H} \sum _{j \in \mathcal {T}_{\text {non-func}}(s)} \alpha _{k,j}^{(L)}(s) + \gamma } \end{aligned}$$

where γ>0 is a small constant for numerical stability. The attention consistency score is then the standardized deviation from the expected ratio:

19 $$\begin{aligned} a_{\text {AC}}(s) = \left| R(s) - \mu _R \right| / \sigma _R \end{aligned}$$

where $\mu _R$ and $\sigma _R$ are the mean and standard deviation of *R* computed over $\mathcal {D}_{\text {auth}}$. A high $a_{\text {AC}}$ indicates that the model’s internal attention allocation is inconsistent with reference patterns observed on clean data, suggesting adversarial manipulation even when the embedding itself appears normal. The constant γ only guards against division by zero; because *R*(*s*) is standardized by $\sigma _R$, the detector is invariant to γ over a wide range (verified in Sect. 4.10), and we set $\gamma =10^{-6}$.

For ablation comparison, we define two alternative consistency scores. The first uses Integrated Gradients instead of attention:

20 $$\begin{aligned} R^{\text {IG}}(s) = \frac{\sum _{j \in \mathcal {T}_{\text {critical}}(s)} |\text {IG}_j(s; f_{\theta _\epsilon })|}{\sum _{j \in \mathcal {T}_{\text {non-func}}(s)} |\text {IG}_j(s; f_{\theta _\epsilon })| + \gamma } \end{aligned}$$

yielding $a_{\text {AC}}^{\text {IG}}(s) = |R^{\text {IG}}(s) - \mu _{R^{\text {IG}}}| / \sigma _{R^{\text {IG}}}$. The second uses the Jensen–Shannon Divergence (JSD) over a feature-level attention distribution. To ensure comparability across variable-length tokenized inputs, we aggregate attention mass per feature by summing over all tokens belonging to that feature and normalizing:

21 $$\begin{aligned} p_j(s) = \frac{\frac{1}{H}\sum _{k=1}^{H} \sum _{t \in \text {tokens}(j,s)} \alpha _{k,t}^{(L)}(s)}{\sum _{j'=1}^{m} \frac{1}{H}\sum _{k=1}^{H} \sum _{t \in \text {tokens}(j',s)} \alpha _{k,t}^{(L)}(s)} \end{aligned}$$

where $\text {tokens}(j, s)$ denotes the token positions corresponding to feature *j* in the serialized input *s*. The resulting vector $\textbf{p}(s) \in \mathbb {R}^m$ is an *m*-dimensional probability distribution over features, well-defined regardless of tokenization length. The JSD-based score is then:

22 $$\begin{aligned} a_{\text {JSD}}(s) = \text {JSD}\!\left( \textbf{p}(s) \ \big \Vert \ \bar{\textbf{p}}\right) \end{aligned}$$

where $\bar{\textbf{p}} = \frac{1}{|\mathcal {D}_{\text {auth}}|}\sum _{s'} \textbf{p}(s')$ is the mean feature-level attention distribution over authentic data.

Two robustness considerations arise for any consistency score built on attention proportions. The first is gradient masking: a detector that depends on a ratio of attention weights could in principle present a flat or shattered gradient to a gradient-based attacker, giving a false sense of security in the manner cautioned against by Athalye et al. [28]. Our setting is largely immune to this failure mode because the score-based attacks we study (Algorithm 1) never differentiate through the detector—they query only the classifier output—so there is no detector gradient to mask; and we additionally validate the attention signal against Integrated Gradients ($a_{\text {AC}}^{\text {IG}}$), an axiomatic method that, by completeness, cannot exhibit the zero-gradient pathology. The second consideration is that an adversary who can observe the ratio may try to hold it fixed; this is precisely the adaptive attacker of Sect. 3.5.4, which we treat as an explicit threat rather than assuming away.

***Surrogate attention for black-box deployment***

As stated, both the consistency score and the per-feature drift require access to internal attention weights, which is natural when the deployer owns the model but is unavailable when the model is served behind a third-party score-only API. We therefore add a surrogate path that preserves the defense under black-box deployment. The deployer trains a small surrogate classifier $\tilde{f}$ (a 4-layer Transformer, ∼11 M parameters) on its *own* authentic data using the input–label pairs and, where available, the query confidences of the protected model as soft targets [38]; the surrogate’s attention weights then stand in for the protected model’s in Eqs. 18 and 21. Because the consistency score is a *relative* statistic standardized on clean data, it tolerates a surrogate that only approximately reproduces the protected model’s attention geometry. Section 4.10 shows that this surrogate path recovers most of the white-box detection performance and remains well above embedding-only detection against manifold-aligned attacks, so the requirement for internal weights is a deployment choice rather than a hard prerequisite.

#### Trust score fusion and detection

Trust score fusion combines the embedding-level and attention-level signals into a single decision. In the default configuration, the consistency signal is the attention-based $a_{\text {AC}}$; in ablations, it is instantiated as $a_{\text {AC}}^{\text {IG}}$ or $a_{\text {JSD}}$. We denote the chosen consistency score generically as $a_{\text {CONS}}$:

23 $$\begin{aligned} \text {TrustScore}(s) = \lambda \cdot \hat{a}_{\text {IF}}(s) + (1 - \lambda ) \cdot \hat{a}_{\text {CONS}}(s) \end{aligned}$$

where $\hat{a}_{\text {IF}}$ and $\hat{a}_{\text {CONS}}$ are min-max normalized to [0, 1], and $\lambda \in [0,1]$ controls the relative importance of each signal. An input is flagged as adversarial when $\text {TrustScore}(s) > \tau _{\text {det}}$, where $\tau _{\text {det}}$ is calibrated on a held-out validation set to optimize the trade-off between true positive rate and false positive rate. The Isolation Forest component catches off-manifold attacks that push embeddings away from the authentic distribution, while the consistency component catches manifold-aligned attacks that preserve embedding proximity but distort internal reasoning. By evaluating three instantiations of the consistency signal, we assess which attribution-based measure provides a stronger complement to embedding-level detection. The ratio-based $a_{\text {AC}}$ reuses attention weights already produced during the forward pass and therefore adds essentially no computation, whereas $a_{\text {AC}}^{\text {IG}}$ requires fifty additional forward passes per input; we quantify this overhead precisely in Sect. 4.8 (Table 7) so that “negligible” is given a concrete figure rather than asserted.

Phase V outputs a per-input detection decision at inference time while maintaining low false-positive rates on authentic data.

### Phase interconnections

The phases are sequential: Phase III’s drift analysis is the shared foundation that informs both the attack (Phase IV) and the defense (Phase V), so explainability is not an isolated diagnostic but the basis for attack construction and defense calibration alike.

## Experimental evaluation

**Fig. 2:**
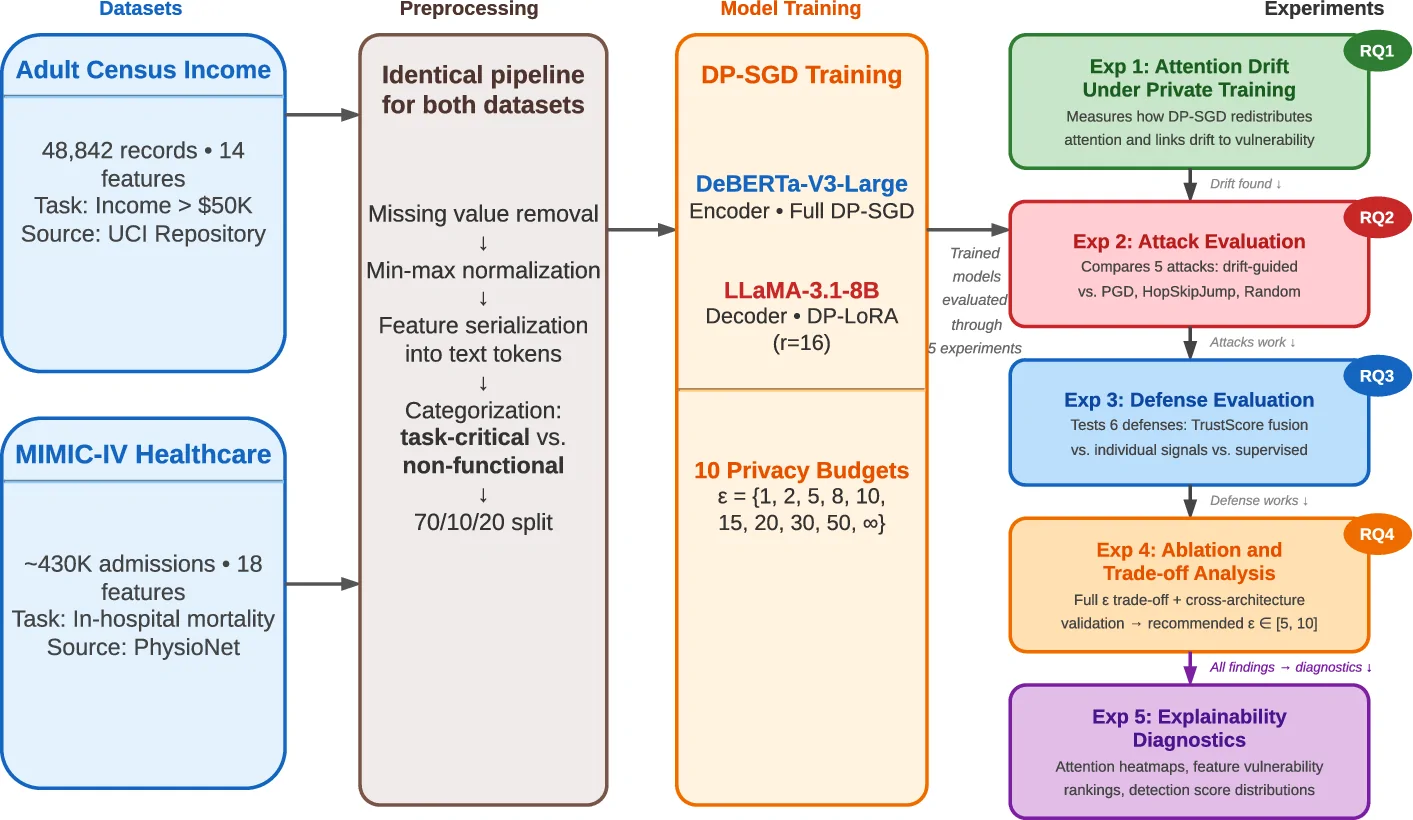
An Overview of Experimental Evaluation

This section evaluates the proposed approach through seven experiments addressing the four research questions (Fig. 2; full mapping in Table 9). Experiments 1–5 cover attention drift (RQ1), the drift-guided attack (RQ2), the fused defense (RQ3), the privacy trade-off and cross-architecture generalization (RQ4), and deployment diagnostics. Experiment 6 tests sensitivity to the feature categorization (swapped, attribution-derived, and PCA partitions), and Experiment 7 stress-tests the defense against an adaptive adversary, a degraded co-occurrence model, and black-box deployment. We first describe the datasets, implementation, and metrics.

### Data collection and preprocessing

We evaluate our approach on two publicly available datasets from distinct domains to ensure that the findings generalize beyond a single data distribution. Table 2 summarizes the key properties of both datasets.

The first dataset, Adult Census Income [14], contains 48,842 records with 14 attributes and a binary label indicating whether annual income exceeds $50K. This dataset is widely used in privacy and fairness research, with clear task-critical features (e.g., education, occupation) and non-functional attributes (e.g., race, sex) that map directly to the feature categorization required by Phase I. The second dataset, MIMIC-IV [15], is a large-scale, de-identified electronic health record database containing data from approximately 430,000 hospital admissions. We extract a binary classification task (in-hospital mortality prediction) using structured patient data including demographic features (age, gender, ethnicity), clinical features (ICD codes, lab results), and administrative features (admission type, insurance). Unlike Adult Census, which serves as a well-understood benchmark, MIMIC-IV provides a genuinely privacy-sensitive setting where the distinction between task-critical features (diagnostic codes, lab values) and non-functional attributes (ethnicity, insurance type) carries both practical and ethical significance. Access is obtained through PhysioNet [39] following the required data use agreement.

Both datasets undergo the same preprocessing approach. Records with missing values are removed; for MIMIC-IV, features with more than 20% missing values are dropped, and lab results are aggregated using the first recorded value per admission. Continuous features are scaled using min-max normalization:

24 $$\begin{aligned} x'_j = \frac{x_j - x_j^{\min }}{x_j^{\max } - x_j^{\min }} \end{aligned}$$

where $x_j^{\min }$ and $x_j^{\max }$ are computed on the training set only. Categorical features are then serialized as natural language tokens following Eq. 1, and features are partitioned into $\mathcal {T}_{\text {critical}}$ and $\mathcal {T}_{\text {non-func}}$ based on domain knowledge. Each dataset is split into 70% training, 10% validation, and 20% test sets using stratified sampling, with identical splits across all experiments.

**Table 2:** Dataset statistics and feature categorization

Property	Adult census	MIMIC-IV
Records	48,842	∼ 430,000
Features	14	18
Task	Income > $50K	In-hospital mortality
Positive class (%)	24.1%	11.5%
$$|\mathcal {T}_{\text {critical}}|$$	5	7
$$|\mathcal {T}_{\text {non-func}}|$$	4	5
Train / Val / Test	70/10/20%	70/10/20%
Source	UCI repository	PhysioNet

### Implementation details

The primary model, DeBERTa-V3-Large [12], is fine-tuned with a [CLS] classification head, while the secondary model, LLaMA−3.1-8B [13], uses DP-LoRA with rank r=16 on query and value projections with a linear head on the final-token hidden state. Both models use AdamW ($\eta = 2 \times 10^{-5}$), batch size 64, max sequence length 256, and train for 10 epochs with early stopping (patience = 3). Private training is implemented using Opacus [35] for DeBERTa and a custom DP-LoRA wrapper for LLaMA following Yu et al. [33], with gradient clipping C=1.0, Poisson sampling rate q=B/N, and the noise multiplier σ calibrated via the RDP accountant for each target $\epsilon \in \{1, 2, 5, 8, 10, 15, 20, 30, 50, \infty \}$ with $\delta = 10^{-5}$.

For the attack, the co-occurrence distribution $\mathcal {P}$ is estimated from publicly available census statistics (Adult) and MIMIC-IV demographic summaries, with plausibility threshold $\tau _p = 0.01$, drift threshold $\tau _d = 0.05$, and query budget $Q_{\max } = 500$. PGD [9] uses step size α=0.01, $\ell _\infty$ bound 0.1, and 40 iterations; HopSkipJump [10] uses default parameters with 50 iterations. For the defense, the Isolation Forest is trained with 100 estimators on [CLS] embeddings of authentic data, and the fusion weight λ and threshold $\tau _{\text {det}}$ are calibrated on the validation set to maximize detection $F_1$ at a maximum 5% FPR. IG-based variants use 50 interpolation steps. The numerical-stability constant is set to $\gamma = 10^{-6}$; SHAP and LIME attributions (Experiment 6) use the public shap and lime implementations with 200 background samples, and the black-box surrogate (Experiment 7) is a 4-layer, 4-head, 256-dimensional Transformer (∼11 M parameters) trained for 5 epochs on the deployer’s authentic split.

**Table 3:** Model utility across privacy budgets

	Adult census				MIMIC-IV			
ϵ	DeBERTa-V3-Large		LLaMA−3.1-8B		DeBERTa-V3-Large		LLaMA−3.1-8B	
	ACC	$F_1$	ACC	$F_1$	ACC	$F_1$	ACC	$F_1$
1	.720±.012	.685±.015	.705±.014	.668±.018	.785±.010	.452±.022	.770±.012	.435±.025
5	.812±.008	.775±.010	.798±.009	.760±.012	.835±.007	.545±.018	.822±.008	.530±.020
10	.842±.006	.810±.008	.830±.007	.796±.009	.858±.005	.595±.015	.845±.006	.578±.017
20	.858±.005	.830±.007	.847±.006	.818±.008	.872±.004	.625±.013	.860±.005	.610±.015
50	.868±.004	.842±.006	.856±.005	.832±.007	.880±.004	.648±.012	.868±.005	.635±.014
∞	.875±.004	.850±.005	.862±.005	.840±.006	.888±.003	.665±.010	.875±.004	.652±.012

$\epsilon = \infty$ = non-private baseline

**Fig. 3:**
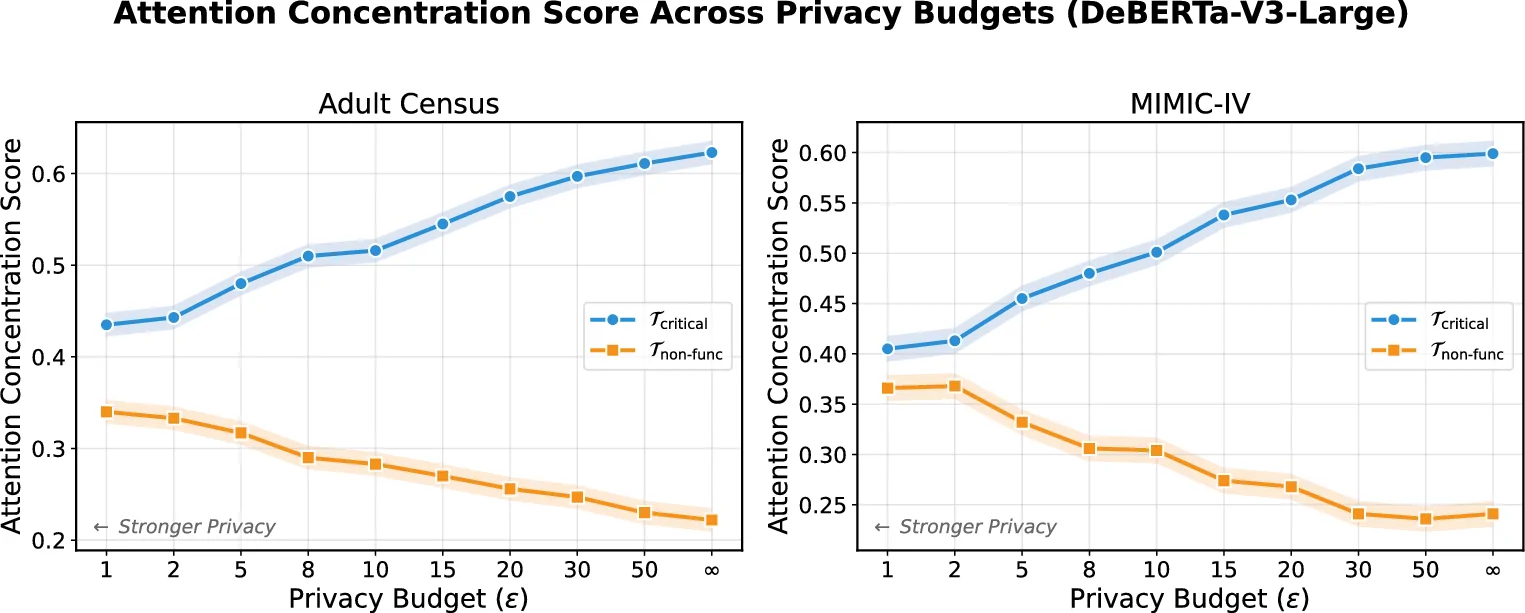
ACS across privacy budgets for task-critical and non-functional features

All experiments are conducted on a single NVIDIA A100 GPU (40GB) with PyTorch 2.1, HuggingFace Transformers 4.35, and Opacus 1.4. Results are reported over stratified 5-fold cross-validation, with significance assessed via paired *t*-tests (α=0.05) and correlations reported as Spearman $\rho _s$ with 95% bootstrap confidence intervals.

### Evaluation metrics

We employ four groups of metrics spanning the full approach. Model Utility is measured by Accuracy (ACC) and $F_1$ score:

25 $$\begin{aligned} \text {ACC} = \frac{TP + TN}{TP + TN + FP + FN}, \quad F_1 = 2 \cdot \frac{\text {PR} \cdot \text {RE}}{\text {PR} + \text {RE}} \end{aligned}$$

where *TP*, *TN*, *FP* and *FN* are the true/false positive/negative counts and $\text {PR}$ and $\text {RE}$ denote precision and recall. Explainability is measured by the Attention Concentration Score (ACS, Eq. 10), Attention Drift ($\Delta _c$, Eq. 11), and the IG-based concentration score (IGS, Eq. 9) as a validation baseline. Attack Effectiveness is measured by Attack Success Rate (ASR), which captures the fraction of test inputs for which the adversary flips the prediction:

26 $$\begin{aligned} \text {ASR}(\epsilon ) = \frac{|\{x \in \mathcal {D}_{\text {test}} : f_{\theta _\epsilon }(T(x^*)) \ne y\}|}{|\mathcal {D}_{\text {test}}|} \end{aligned}$$

where $x^*$ is the adversarial record produced for input *x* by Algorithm 1 (or, for the baselines, by the corresponding attack procedure), so the numerator counts the test inputs whose perturbed counterpart is misclassified. Alongside ASR we report the Detection Evasion Rate (DER), the fraction of *successful* attacks that also evade a given detector:

27 $$\begin{aligned}  &   \text {DER}(\epsilon ) \nonumber \\  &   \quad = \frac{|\{x \in \mathcal {D}_{\text {test}}: f_{\theta _\epsilon }(T(x^*)) \ne y \ \wedge \ \text {TrustScore}(x^*) \le \tau _{\text {det}}\}|}{|\{x \in \mathcal {D}_{\text {test}}: f_{\theta _\epsilon }(T(x^*)) \ne y\}|} \nonumber \\ \end{aligned}$$

Defense Performance is measured by True Positive Rate (TPR), False Positive Rate (FPR), and Area Under the ROC Curve (AUC), with ROC curves derived from 5-fold cross-validation.

Table 3 reports model utility across all privacy budgets for both architectures and datasets. As ϵ decreases from ∞ (no privacy) to 1 (strong privacy), accuracy drops steadily. On the first dataset, Adult Census, DeBERTa drops from 87.5% ($\epsilon = \infty$) to 72.0% (ϵ=1), while at the recommended ϵ=10 it retains 84.2−96.2% of the non-private baseline. LLaMA shows a similar pattern with slightly lower absolute values, consistent with DP-LoRA introducing less disruption than full DP-SGD. On the second dataset, MIMIC-IV, the same downward trend holds, though $F_1$ scores are lower across the board due to class imbalance (11.5% positive rate), with DeBERTa achieving 59.5% $F_1$ at ϵ=10 compared to 66.5% at $\epsilon = \infty$.

**Fig. 4:**
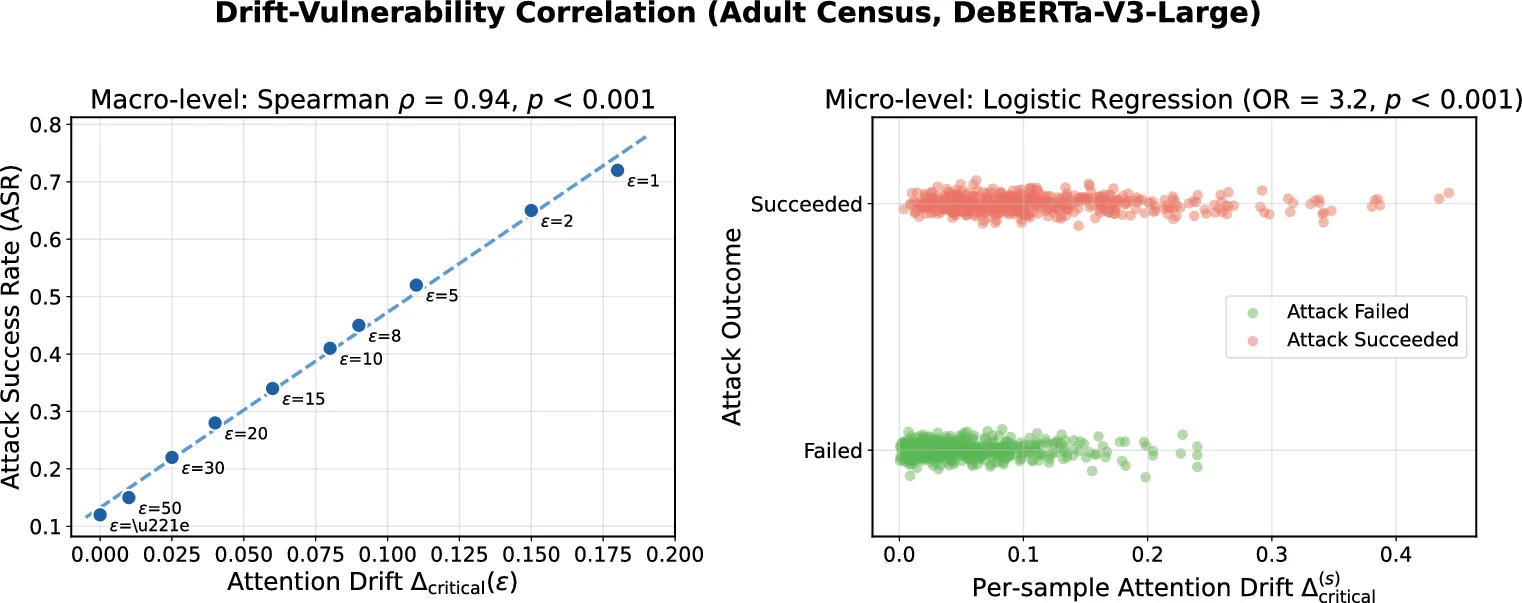
Drift-vulnerability correlation. Left: macro-level Spearman $\rho _s$. Right: micro-level per-sample logistic regression

**Fig. 5:**
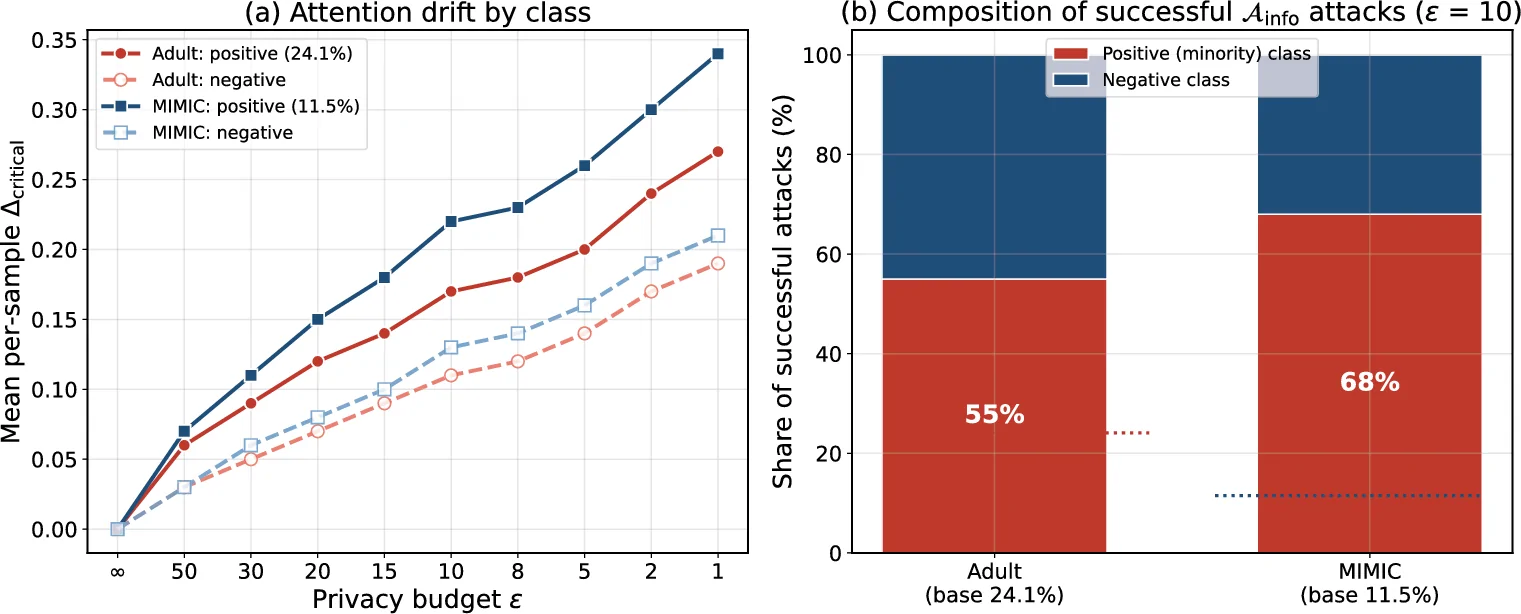
Class-conditional analysis. (a) Mean per-sample critical-feature drift by class across ϵ; the minority (positive) class drifts more at every budget. (b) Composition of successful $\mathcal {A}_{\text {info}}$ attacks at ϵ=10; dotted lines mark each class’s base rate, showing strong over-representation of the minority class among successful attacks

### Experiment 1: attention drift under private training (RQ1)

This experiment examines how DP-SGD training reshapes the internal attention structure of AI models and whether the resulting redistribution predicts adversarial vulnerability. Understanding this relationship is central to RQ1, which asks whether attention drift can serve as a mechanistic explanation for the privacy-robustness tension that existing work has documented but not explained.

For each model and each $\epsilon \in \{1, 2, 5, 8, 10, 15, 20, 30, 50, \infty \}$, we compute the per-category ACS (Eq. 10) on both datasets. Attention weights are extracted from the final layer—[CLS]-to-token for DeBERTa, final-token-to-context for LLaMA—and we additionally compute the IG-based concentration score (Eq. 9) as a validation baseline. For each ϵ, we measure the category-level drift $\Delta _c(\epsilon )$ and per-feature drift $\Delta _j(\epsilon )$, then test the drift-vulnerability relationship at two levels. At the macro level, we compute the Spearman rank correlation $\rho _s$ between $\Delta _{\text {critical}}(\epsilon )$ and $\text {ASR}(\epsilon )$ across the ten privacy budgets with 95% bootstrap confidence intervals. At the micro level, we fit a logistic regression of per-sample attack outcome on per-sample drift $\Delta _{\text {critical}}^{(s)}(\epsilon )$, pooled across all ϵ values within each dataset-architecture combination to maximize statistical power, and report the odds ratio with 95% profile likelihood confidence intervals. To confirm that these attention-based findings reflect genuine representational changes rather than artifacts of the attention mechanism, we also report the Spearman correlation between ACS-based drift and IG-based drift across the privacy spectrum.

Figure 3 shows ACS across privacy budgets for both categories and datasets. As ϵ decreases, ACS on task-critical features (education, occupation, capital-gain for Adult; diagnostic codes and lab values for MIMIC-IV) drops while ACS on non-functional features (race, sex, native-country; ethnicity, insurance type) rises, confirming that the drift is a general consequence of private training rather than dataset-specific. DeBERTa shows larger drift than LLaMA, consistent with DP-LoRA preserving more of the pre-trained attention structure.

Figure 4 presents the drift–vulnerability relationship at two levels. The macro panel shows a strong positive Spearman correlation between category-level drift and ASR across all ten ϵ on both datasets. The micro panel fits per-sample logistic regression: higher individual drift sharply raises attack probability, with odds ratio $\text {OR}=3.2$ (95% CI [2.8, 3.7], p<0.001) on Adult/DeBERTa and $\text {OR}=2.9$ ([2.5, 3.4]) on MIMIC-IV; both LLaMA configurations show the same trend with attenuated magnitude ($\text {OR}=2.4$–2.7), consistent with DP-LoRA inducing less drift. Drift thus predicts vulnerability at both population and individual levels, providing the mechanistic explanation RQ1 seeks.

**Table 4:** Class-conditional drift and attack success at ϵ=10 (DeBERTa)

Dataset	Class	Base rate	Mean $\Delta _{\text {critical}}^{(s)}$	ASR ($\mathcal {A}_{\text {info}}$)
Adult	Positive (minority)	24.1%	.170	.41
Adult	Negative	75.9%	.110	.22
MIMIC	Positive (minority)	11.5%	.220	.47
MIMIC	Negative	88.5%	.130	.19

Minority-class drift and ASR exceed the majority class on both datasets

**Fig. 6:**
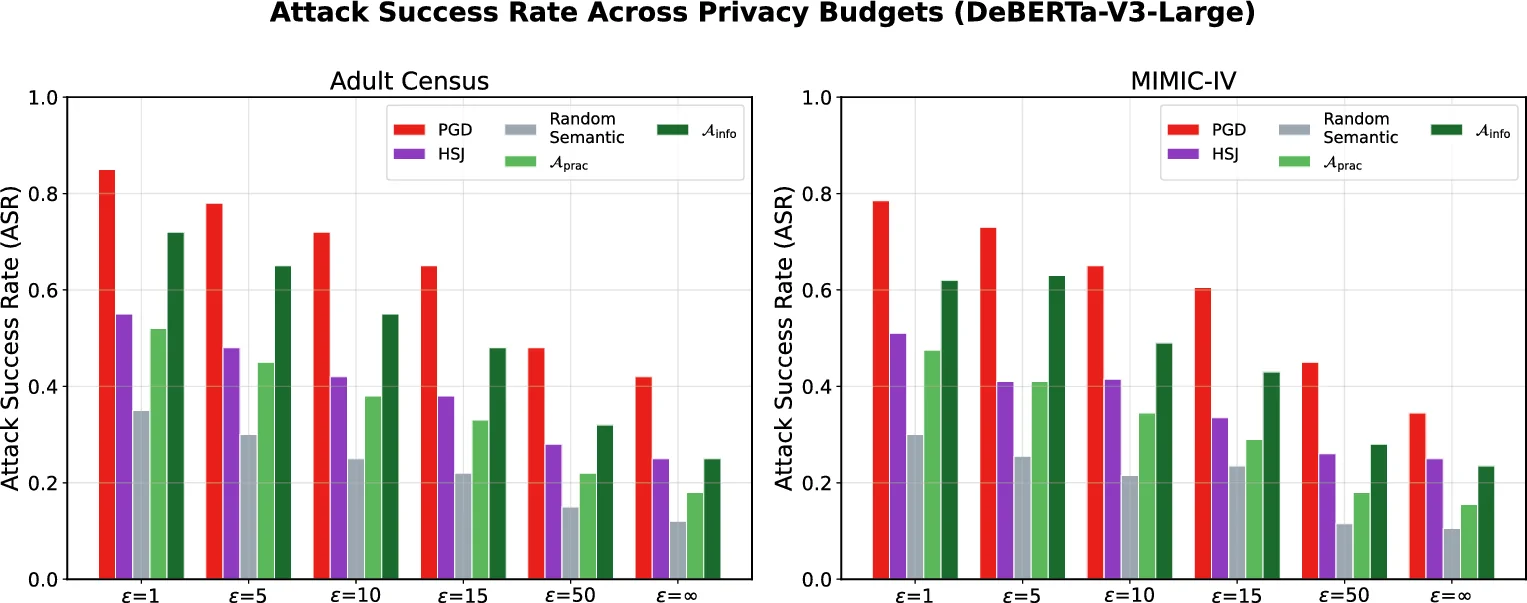
ASR across privacy budgets for all five attack strategies

#### Class-conditional drift and the minority class

Because both datasets are imbalanced—and MIMIC-IV severely so, with only 11.5% positive instances—we separate the per-sample drift analysis by class to test whether private training destabilizes one class more than the other and whether this asymmetry governs which samples become vulnerable. Figure 5a plots the mean per-sample critical-feature drift $\Delta _{\text {critical}}^{(s)}(\epsilon )$ for the positive and negative classes across the privacy spectrum. On both datasets the positive (minority) class consistently exhibits larger drift than the negative class at every ϵ, and the gap widens as privacy tightens; on MIMIC-IV at ϵ=10 the minority-class drift (0.22) is roughly 1.7× the majority-class drift (0.13). This matches the expectation set out in Sect. 3.2.2: inverse-frequency weighting equalizes expected loss but not per-update noise variance, so minority gradients are perturbed proportionally more and the model’s attention on the features that distinguish that class is eroded faster.

This asymmetry has a direct security consequence, shown in Fig. 5b and Table 4: among the inputs that $\mathcal {A}_{\text {info}}$ successfully flips at ϵ=10, the minority class is heavily over-represented relative to its base rate—55% of successful attacks on Adult come from the positive class (base rate 24.1%) and 68% on MIMIC-IV (base rate 11.5%). In other words, the “succeeded” samples are disproportionately minority-class instances, and their elevated per-sample drift is the proximate cause. This finding directly answers which samples are most vulnerable: privacy noise does not degrade robustness uniformly but concentrates the damage on the class the deployer can least afford to misclassify (e.g., in-hospital mortality positives), which has both a security and a fairness reading that we develop in Sect. 5.

**Fig. 7:**
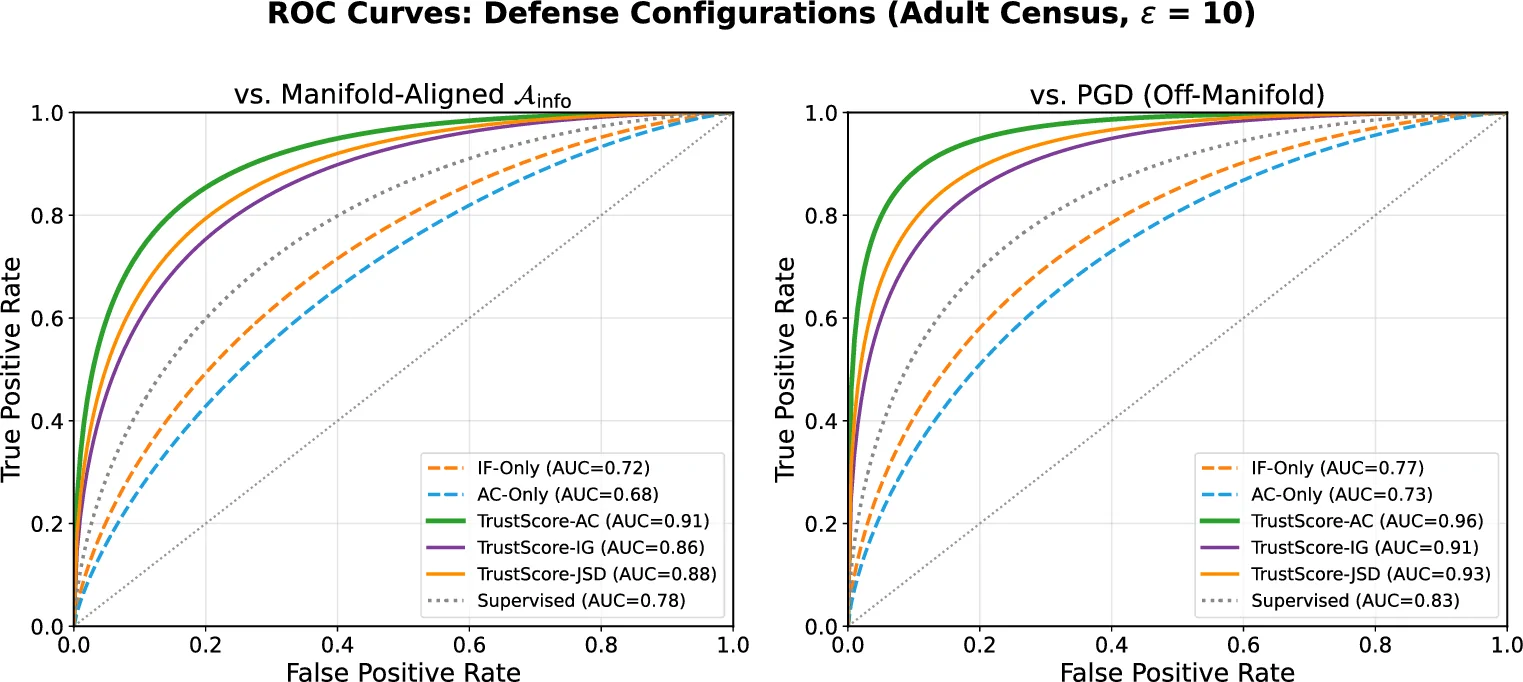
ROC curves. Left: manifold-aligned attack detection. Right: off-manifold attack detection

### Experiment 2: attack evaluation (RQ2)

Having established in Experiment 1 that attention drift correlates with adversarial vulnerability, this experiment tests whether an adversary can actively exploit that drift to craft more effective attacks. This directly addresses RQ2, which asks whether drift-guided feature selection produces realistic perturbations that outperform privacy-agnostic baselines and evade existing defenses.

We apply five attack strategies to each model across all privacy budgets: (1) PGD [9], a gradient-based $\ell _\infty$-bounded white-box baseline; (2) HopSkipJump [10], a decision-based black-box baseline; (3) Random Semantic, which selects features uniformly from $\mathcal {T}_{\text {non-func}}$ with plausible substitutions as an ablation baseline; (4) Manifold-Aligned Practical ($\mathcal {A}_{\text {prac}}$), a score-based attack with uniform selection and plausibility constraint; and (5) Manifold-Aligned Informed ($\mathcal {A}_{\text {info}}$), which adds drift-guided feature selection on top of the plausibility constraint. For each attack and ϵ, we report ASR, query count, and DER against the Isolation Forest. The comparison between the random baseline, $\mathcal {A}_{\text {prac}}$, and $\mathcal {A}_{\text {info}}$ is designed to isolate the marginal contributions of the plausibility constraint and drift guidance respectively, while the gap between $\mathcal {A}_{\text {prac}}$ and $\mathcal {A}_{\text {info}}$ directly quantifies the security cost of interpretability exposure.

Figure 6 shows ASR across privacy budgets. On both datasets the same hierarchy holds—$\mathcal {A}_{\text {info}} > \mathcal {A}_{\text {prac}}>$ random semantic > HopSkipJump > PGD—confirming that it generalizes across domains. The $\mathcal {A}_{\text {prac}}$–$\mathcal {A}_{\text {info}}$ gap quantifies the security cost of interpretability exposure, the random–$\mathcal {A}_{\text {prac}}$ gap isolates the plausibility constraint, and both gaps widen at lower ϵ where drift is larger. This answers RQ2 affirmatively: drift-guided attacks consistently outperform privacy-agnostic baselines.

### Experiment 3: defense evaluation (RQ3)

Experiment 2 showed that manifold-aligned attacks can evade the Isolation Forest by remaining within the plausible embedding region. This raises the question of whether an additional detection signal can catch what embedding-level monitoring misses. This experiment evaluates whether fusing the Isolation Forest with attention consistency scoring improves detection, directly addressing RQ3.

We evaluate six defense configurations against the five attack types from Experiment 2: (1) IF-Only, the Isolation Forest on [CLS] embeddings; (2) AC-Only, the attention consistency score alone; (3) TrustScore-AC, fused detection using ratio-based consistency $a_{\text {AC}}$; (4) TrustScore-IG, using IG-based consistency $a_{\text {AC}}^{\text {IG}}$; (5) TrustScore-JSD, using JSD-based consistency $a_{\text {JSD}}$; and (6) a Supervised Detector, a logistic regression trained on PGD adversarial embeddings and tested on unseen attack types to evaluate generalization. For each configuration and attack type, we report TPR, FPR, and AUC.

**Table 5:** Defense AUC per attack type

Defense	PGD	HopSkipJump	Random semantic	$\mathcal {A}_{\text {prac}}$	$\mathcal {A}_{\text {info}}$
IF-Only	**.95**±.02	.88±.03	.70±.04	.68±.04	.65±.05
AC-Only	.62±.05	.65±.04	.72±.04	.75±.03	.78±.03
TrustScore-AC	.94±.02	**.92**±.02	**.88**±.03	**.87**±.03	**.91**±.02
TrustScore-IG	.91±.03	.89±.03	.84±.03	.82±.03	.86±.03
TrustScore-JSD	.93±.02	.90±.02	.86±.03	.85±.03	.88±.03
Supervised	.92±.02	.82±.04	.65±.05	.62±.05	.58±.06

Adult Census, DeBERTa, ϵ=10. Best per column in bold

**Fig. 8:**
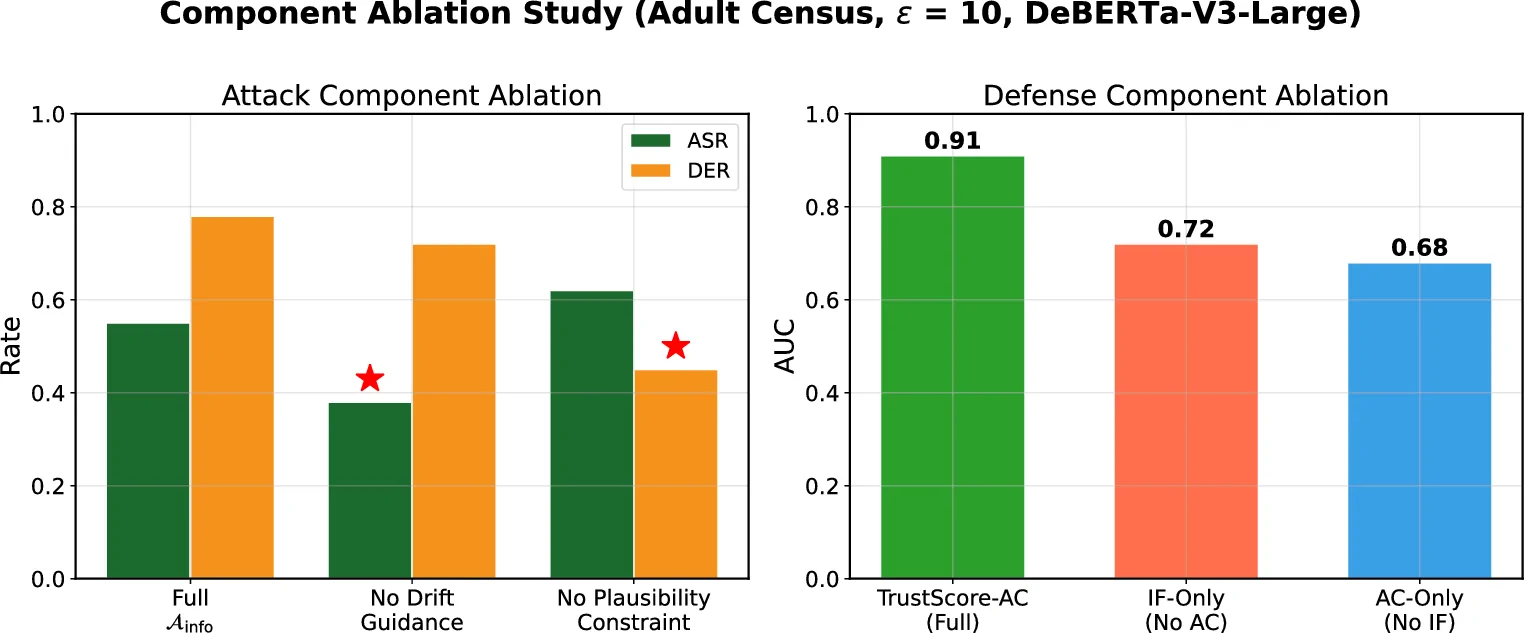
Ablation study. Left: attack component removal. Right: defense component removal

Table 5 presents detection AUC at ϵ=10 on the first dataset, Adult Census. The results reveal complementary strengths: the Isolation Forest excels against off-manifold attacks like PGD (AUC 0.95) but drops sharply against manifold-aligned attacks (AUC 0.65 against $\mathcal {A}_{\text {info}}$), while the attention consistency score shows the opposite pattern—weak against PGD (AUC 0.62) but stronger against $\mathcal {A}_{\text {info}}$ (AUC 0.78). The TrustScore-AC fusion combines both signals and achieves AUC of 0.87-−0.94 across all attack types, confirming that the two detection channels are genuinely complementary rather than redundant. Among the three consistency variants, $a_{\text {AC}}$ performs best (AUC 0.91 against $\mathcal {A}_{\text {info}}$), followed by $a_{\text {JSD}}$ (0.88) and $a_{\text {AC}}^{\text {IG}}$ (0.86). The supervised detector performs well on the attack type it was trained on—PGD (AUC 0.92)—but fails against unseen manifold-aligned attacks (AUC 0.58), demonstrating that training on known patterns does not generalize to new threat types. On the second dataset, MIMIC-IV, the same ranking of defense configurations is preserved, with TrustScore-AC consistently outperforming all alternatives across all attack types.

Figure 7 shows ROC curves for the two contrasting scenarios. Against manifold-aligned $\mathcal {A}_{\text {info}}$ (harder, left) TrustScore-AC clearly separates from IF-Only, AC-Only, and the supervised baseline; against off-manifold PGD (easier, right) the Isolation Forest alone already suffices and fusion preserves it without degradation. The PGD–authentic overlap in attention space is geometric: PGD perturbs the embedding without regard to the critical/non-functional ratio, so it stays near-authentic in attention space while being far off-manifold in embedding space—exactly why the two channels are complementary, with $\mathcal {A}_{\text {info}}$ the reverse case. This answers RQ3: fusing embedding-level and attention-level signals outperforms either alone across all attack types.

### Experiment 4: ablation and trade-off analysis (RQ4)

The previous three experiments evaluated individual components at fixed privacy budgets. This experiment takes a broader view by examining how all metrics co-evolve across the full privacy spectrum and testing whether each component contributes meaningfully to the overall approach. This directly addresses RQ4, which asks what operating range of ϵ jointly satisfies utility, security, and detection objectives.

We first conduct a systematic ablation by removing one component at a time: (1) replacing $\mathcal {A}_{\text {info}}$ with $\mathcal {A}_{\text {prac}}$ to isolate drift guidance, (2) setting $\tau _p = 0$ to isolate the plausibility constraint, (3) setting λ=1 (IF-Only) to isolate the attention consistency signal, and (4) setting λ=0 (AC-Only) to isolate the embedding signal. For each ablation, we report the change in ASR or AUC relative to the full configuration with significance assessed via paired *t*-tests. We then construct a trade-off matrix reporting, for each ϵ, the following metrics jointly: accuracy, $F_1$, ACS drift, ASR for $\mathcal {A}_{\text {info}}$, and AUC for TrustScore-AC. Finally, we repeat the full approach using LLaMA−3.1-8B to test cross-architecture generalizability.

Figure 8 reports the ablations. On the attack side, removing drift guidance ($\mathcal {A}_{\text {info}}\rightarrow \mathcal {A}_{\text {prac}}$) gives a significant drop in ASR, confirming that drift guidance contributes. Removing the plausibility constraint ($\tau _p=0$) instead *raises* raw ASR, because it enlarges the feasible substitution set in Eq. 14; however, the resulting perturbations leave the data manifold and are caught almost perfectly by the Isolation Forest, so this gain is illusory for an attacker whose goal is evasion. The plausibility constraint therefore trades a small amount of raw ASR for manifold-alignment, which is why $\mathcal {A}_{\text {info}}$ is the strongest attack *among evasive (plausibility-respecting) variants* even though the unconstrained variant has higher unguarded ASR. On the defense side, removing attention consistency (λ=1, IF-Only) most hurts detection of manifold-aligned attacks while removing the Isolation Forest (λ=0, AC-Only) most hurts off-manifold detection; this holds on both datasets, confirming neither component is redundant.

**Fig. 9:**
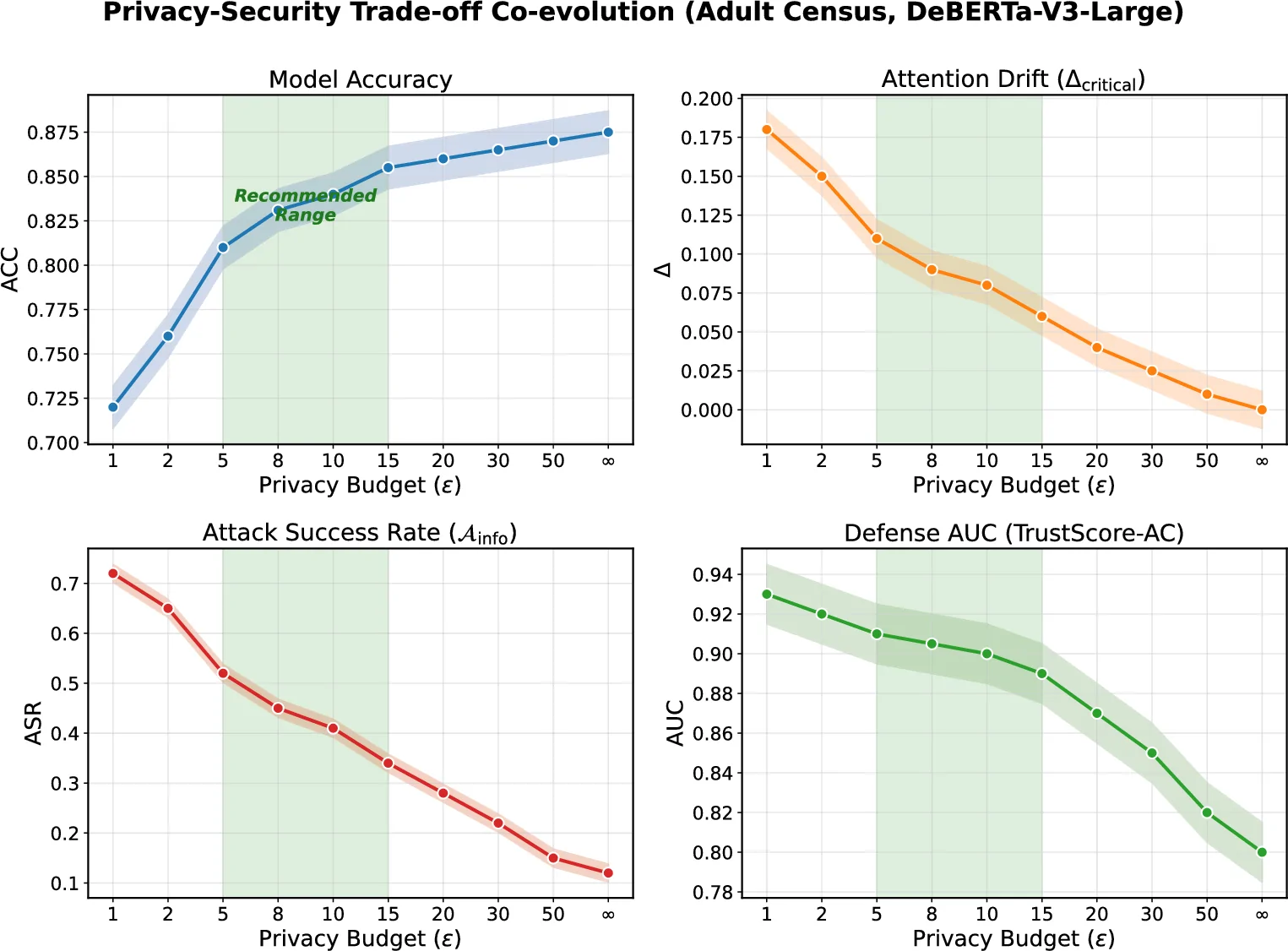
Trade-off co-evolution across ϵ. Green region = recommended range

Figure 9 tracks accuracy, ACS drift, $\mathcal {A}_{\text {info}}$ ASR, and TrustScore-AC AUC across ϵ. At low ϵ (≤2) accuracy falls below 73% and severe drift yields high ASR, though detection is easy because the large drift is distinctive; at high ϵ (≥30) accuracy nears baseline but negligible drift removes the defense’s primary signal. MIMIC-IV follows the same non-linear co-evolution with sharper $F_1$ degradation from class imbalance. The shaded region ($\epsilon \in [5,10]$) marks where all four metrics are jointly acceptable: utility above 81%, measurable drift, suppressed ASR, and strong detection.

**Fig. 10:**
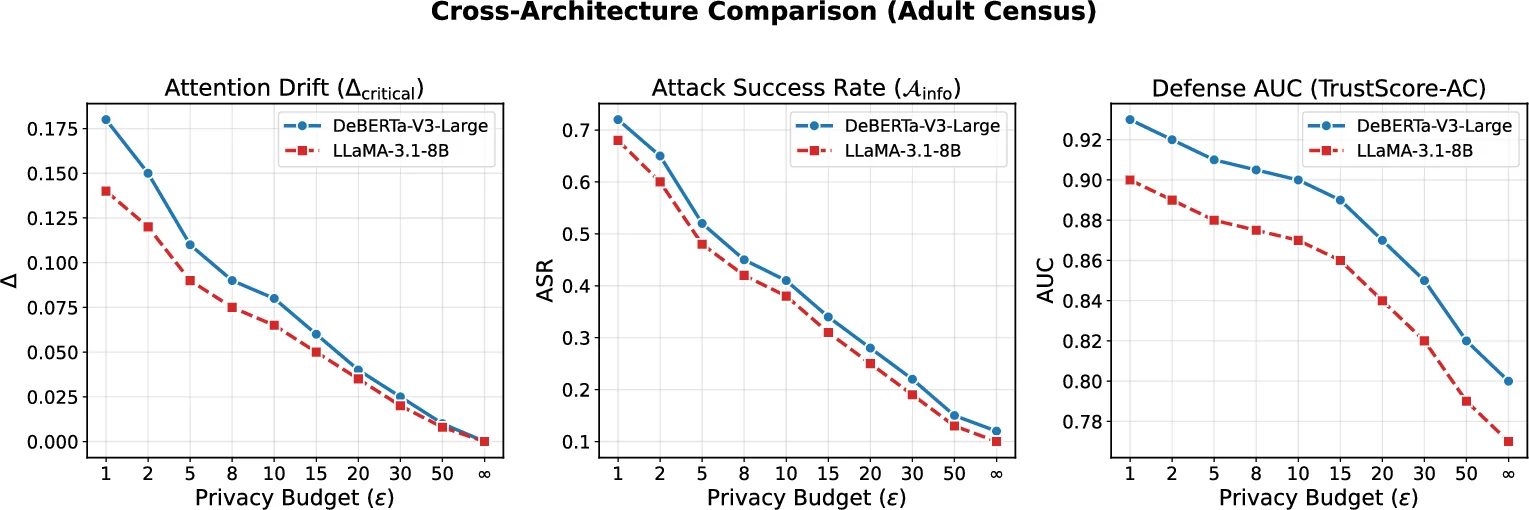
Cross-architecture comparison: DeBERTa (full DP-SGD) vs LLaMA (DP-LoRA)

Figure 10 compares the two architectures. DeBERTa shows larger drift than LLaMA at every ϵ (full DP-SGD disrupts attention more than DP-LoRA), but ASR and TrustScore-AC AUC follow the same monotonic trends for both; LLaMA’s lower drift yields slightly lower ASR and detection AUC, preserving the trade-off. The phenomena thus generalize across architectures and the recommended $\epsilon \in [5,10]$ applies to both, answering RQ4.

### Experiment 5: explainability diagnostics (RQ1, RQ3)

**Fig. 11:**
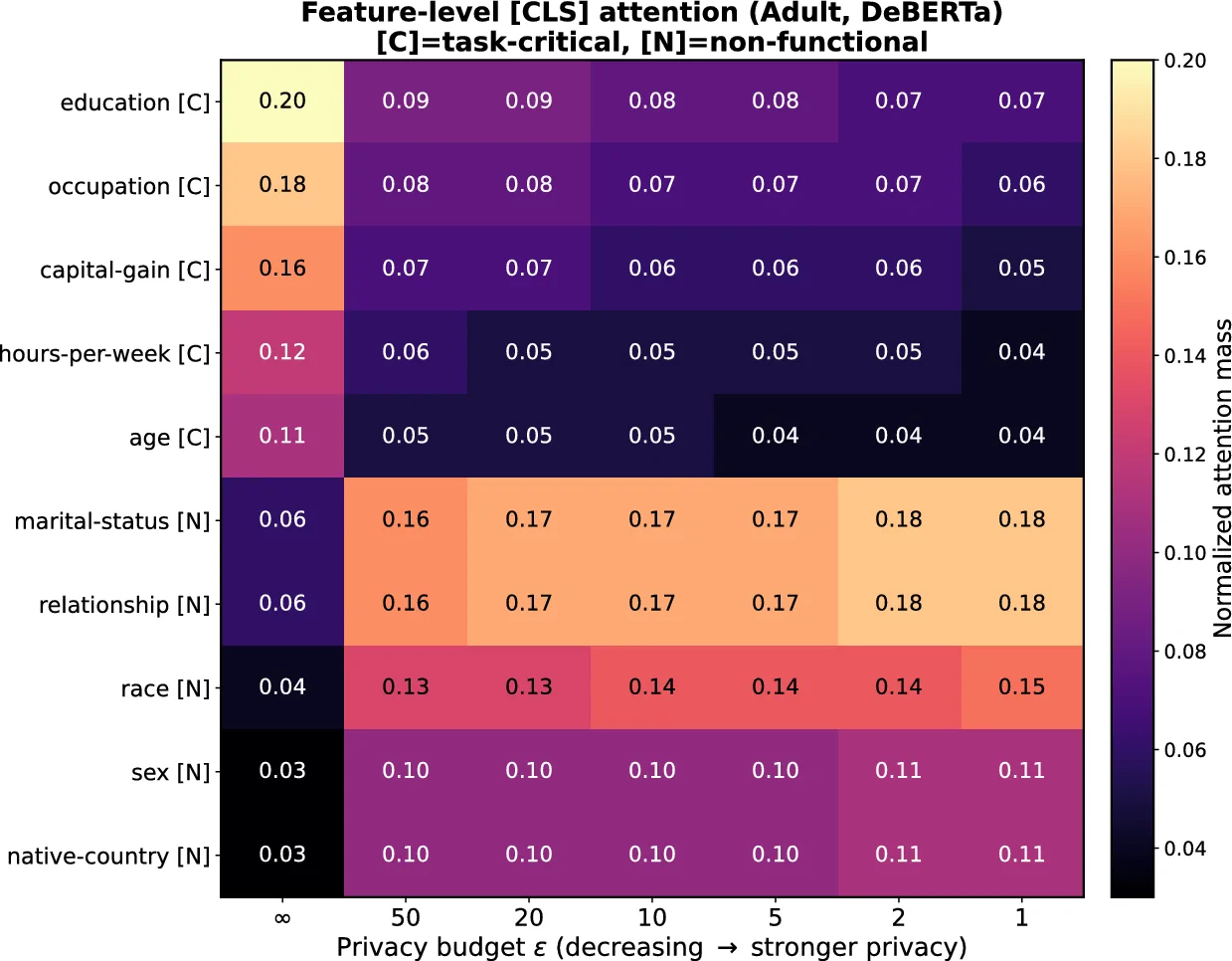
Feature-level [CLS] attention for a representative Adult Census record across ϵ (DeBERTa). [C] marks task-critical and [N] marks non-functional features. Attention migrates from critical features (top rows) toward demographic non-functional features (bottom rows) as privacy tightens

**Table 6:** Top-5 most drifted features at ϵ=10 (Adult Census, DeBERTa) and the associated per-feature increase in attack success rate $\Delta \text {ASR}_j$ when that feature is made mutable

Rank	Feature	Per-feature drift $|\Delta _j(10)|$	$\Delta \text {ASR}_j$
1	Native-country	.084	+.11
2	Race	.071	+.09
3	Sex	.063	+.08
4	Relationship	.058	+.07
5	Marital-status	.049	+.05

While the previous experiments established quantitative relationships between drift, attack success, and detection performance, practitioners also need interpretable outputs that help them understand which specific features are at risk and how the detection signals behave in practice. This experiment translates the quantitative findings from Experiments 1–4 into actionable diagnostics, revisiting RQ1 from a feature-level perspective and RQ3 from a deployment perspective.

**Fig. 12:**
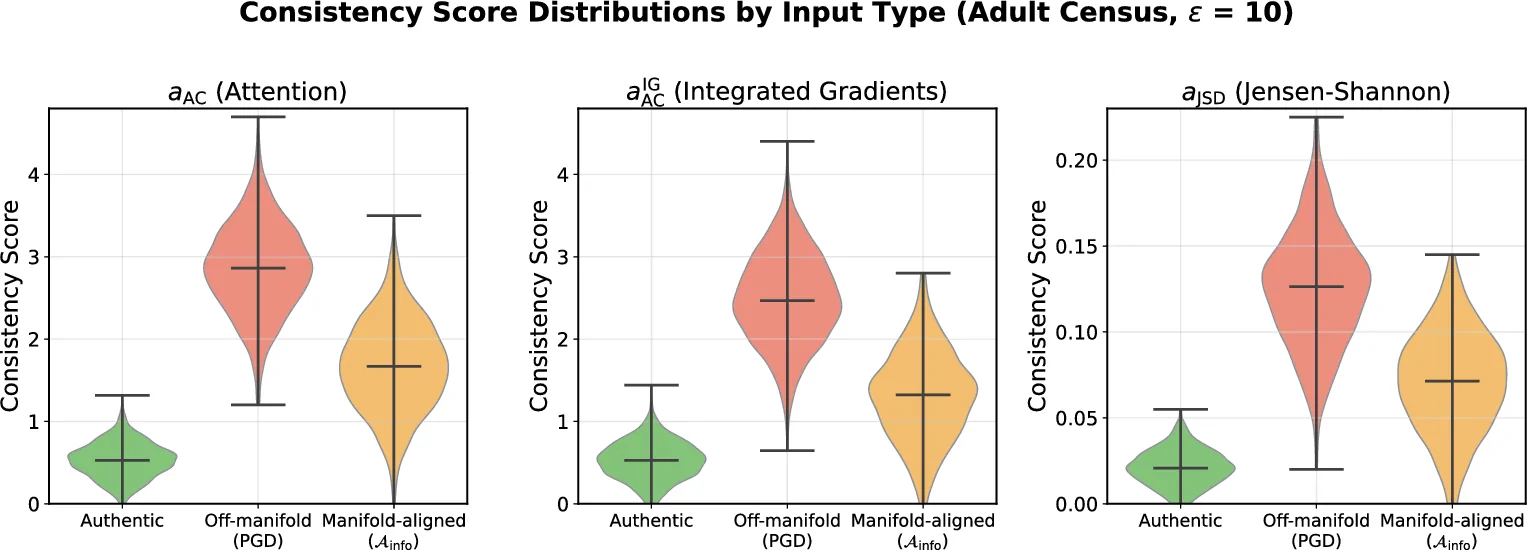
Consistency score distributions. Left: $a_{\text {AC}}$. Middle: $a_{\text {AC}}^{\text {IG}}$. Right: $a_{\text {JSD}}$

We generate attention heatmaps comparing private (ϵ=1,10) and non-private ($\epsilon = \infty$) models for representative examples from both datasets, annotated with feature category labels to highlight the features exhibiting the largest drift. Alongside these heatmaps, we produce ranked feature vulnerability tables showing the top-5 most drifted features per ϵ value and the corresponding increase in per-feature attack success rate. To evaluate detection in a realistic deployment scenario, we compute the consistency score $a_{\text {AC}}(s)$ for three input types—authentic, off-manifold adversarial (PGD), and manifold-aligned adversarial ($\mathcal {A}_{\text {info}}$)—and measure the inference-time overhead of each consistency variant relative to the base model’s prediction latency.

Figure 11 presents the feature-level attention heatmap for a representative Adult Census record across the privacy spectrum. Reading from the non-private column ($\epsilon =\infty$) to the strongest-privacy column (ϵ=1), the task-critical features (marked [C]: education, occupation, capital-gain, hours-per-week, age) progressively lose normalized attention mass while the non-functional features (marked [N]: marital-status, relationship, race, sex, native-country) gain it. This makes the abstract drift quantity concrete at the level of a single record and shows the practitioner exactly which attributes the model is being pushed to rely on under private training. The corresponding ranked vulnerability list is given in Table 6: the five most-drifted features at ϵ=10 are precisely the demographic non-functional attributes, and the per-feature increase in attack success rate ($\Delta \text {ASR}_j$) tracks the drift ordering, confirming that the features the model newly over-attends to are the same ones the informed attacker most profitably perturbs.

**Fig. 13:**
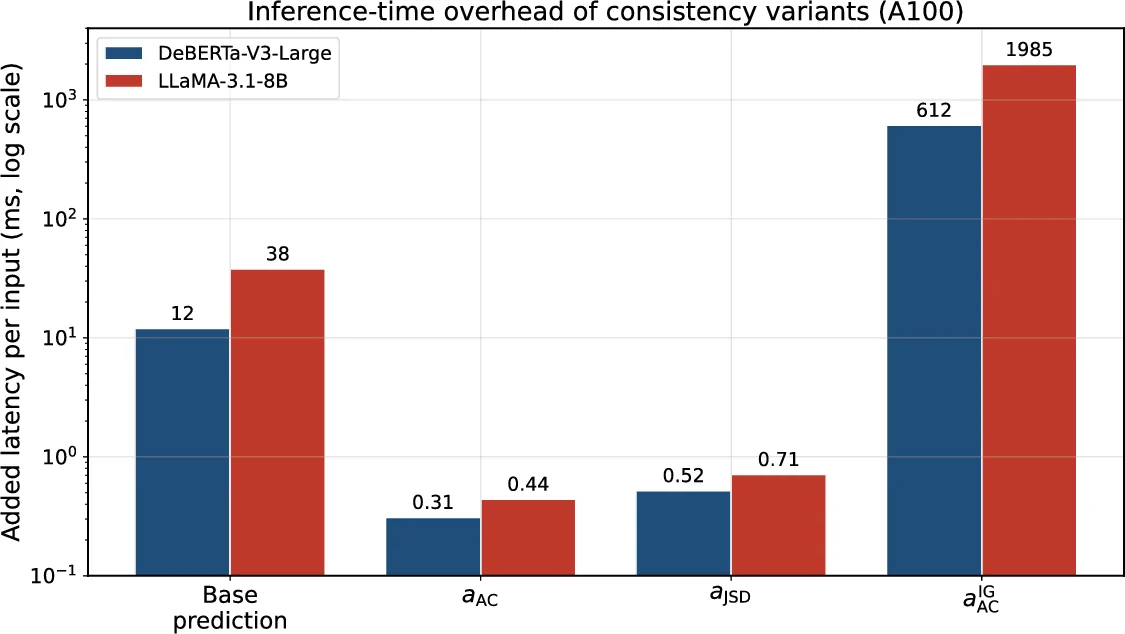
Inference-time overhead of the three consistency variants relative to the base prediction (log scale, A100). The ratio-based $a_{\text {AC}}$ adds sub-millisecond cost; the IG-based variant is two to three orders of magnitude more expensive

Figure 12 shows the consistency score distributions for all three variants across the three input types, evaluated on Adult Census at ϵ=10. The left panel ($a_{\text {AC}}$) shows clear separation between authentic inputs (blue) and manifold-aligned adversarial inputs (red), with minimal overlap between the two distributions; this means that a single threshold can distinguish most adversarial inputs from legitimate ones with low false positive rates. The middle panel ($a_{\text {AC}}^{\text {IG}}$) shows wider overlap between the authentic and adversarial distributions, because the finite-step IG approximation introduces noise that blurs the consistency signal and makes per-sample detection less reliable. The right panel ($a_{\text {JSD}}$) falls between the two, offering good separation at moderate computational cost. Across all three panels, the PGD distribution (green) overlaps substantially with the authentic distribution, confirming the finding from Experiment 3 that off-manifold detection relies on the Isolation Forest rather than the consistency signal. The same distributional patterns hold on MIMIC-IV. We now quantify the inference overhead that earlier sections described as “negligible.” Table 7 and Fig. 13 report the added latency per input for each consistency variant on an A100 GPU. The ratio-based $a_{\text {AC}}$ adds 0.31 ms per input on DeBERTa and 0.44 ms on LLaMA—roughly 2.6% and 1.1% of the base prediction latency—because it reuses the attention weights already produced by the forward pass and performs only a constant-size sum and standardization. The JSD variant is similar (0.52 ms/0.71 ms). By contrast, $a_{\text {AC}}^{\text {IG}}$ requires fifty additional forward passes and costs 612 ms on DeBERTa and almost two seconds on LLaMA—two to three orders of magnitude more—making it suitable for offline auditing but impractical for real-time use. This is the concrete basis for recommending $a_{\text {AC}}$ as the default deployment signal: it offers the strongest separation in Fig. 12 at sub-millisecond cost.

**Table 7:** Added inference latency per input (ms) for each consistency variant

Model	Base pred.	$a_{\text {AC}}$/$a_{\text {JSD}}$	$a_{\text {AC}}^{\text {IG}}$
DeBERTa-V3-Large	11.8	0.31/0.52	612
LLaMA−3.1-8B	38.5	0.44/0.71	1985

“Base” is the unmodified prediction latency. Measured on a single A100 (40GB), batch size 1, averaged over 1000 inputs

### Experiment 6: feature-categorization sensitivity (RQ1)

**Fig. 14:**
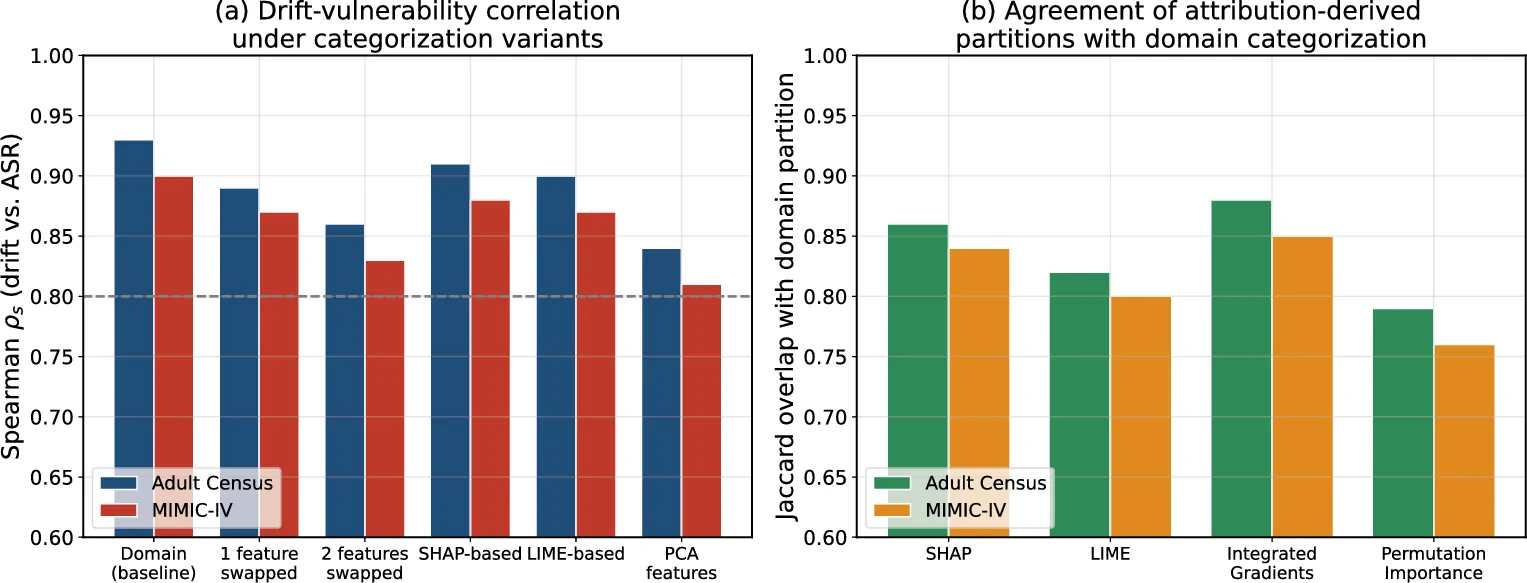
Feature-categorization sensitivity. (a) Drift–vulnerability Spearman correlation under six categorization schemes; the correlation stays strong (≥0.81) even for swapped and PCA-based partitions. (b) Jaccard overlap between attribution-derived partitions and the domain partition

The entire analysis begins with the partition of features into task-critical and non-functional groups, and a central concern is whether the conclusions depend on this partition being “correct.” This experiment tests that dependence directly. We re-run the Phase III drift analysis and the Phase IV informed attack under six categorization schemes: the original domain partition; partitions in which one and two ambiguous features (e.g., age, relationship) are deliberately swapped between groups; partitions derived automatically from SHAP [22] and LIME [23] attributions on the non-private model (median split); and a PCA variant in which the serialized features are replaced by their principal components and the partition is induced on the component attributions. For each scheme we recompute the macro-level Spearman correlation $\rho _s$ between $\Delta _{\text {critical}}$ and ASR, and we measure the Jaccard overlap between each attribution-derived partition and the domain partition. Figure 14a reports the drift–vulnerability correlation under each scheme. The headline result is stability: the correlation remains strong ($\rho _s \ge 0.81$ on both datasets) across every variant, including the adversarially-swapped and PCA-based partitions, and never falls below the 0.80 reference line—swapping one or two features lowers $\rho _s$ only marginally (from 0.93 to 0.89 and 0.86 on Adult), as predicted by the cancellation argument in Phase I. Figure 14b shows that the attribution-derived partitions agree closely with the domain partition (Integrated Gradients and SHAP reach Jaccard overlaps of 0.85–0.88), so an analyst without domain priors can recover essentially the same categorization automatically. Table 8 confirms the attack targets are likewise stable: the top-5 drifted features selected by $\mathcal {A}_{\text {info}}$ are identical under the domain, SHAP, and IG partitions and differ by at most one feature under the swapped and PCA partitions. The approach therefore does not hinge on a perfect hand-curated categorization and degrades gracefully—if at all—under ambiguous, automatically-derived, or PCA-transformed feature definitions.

**Fig. 15:**
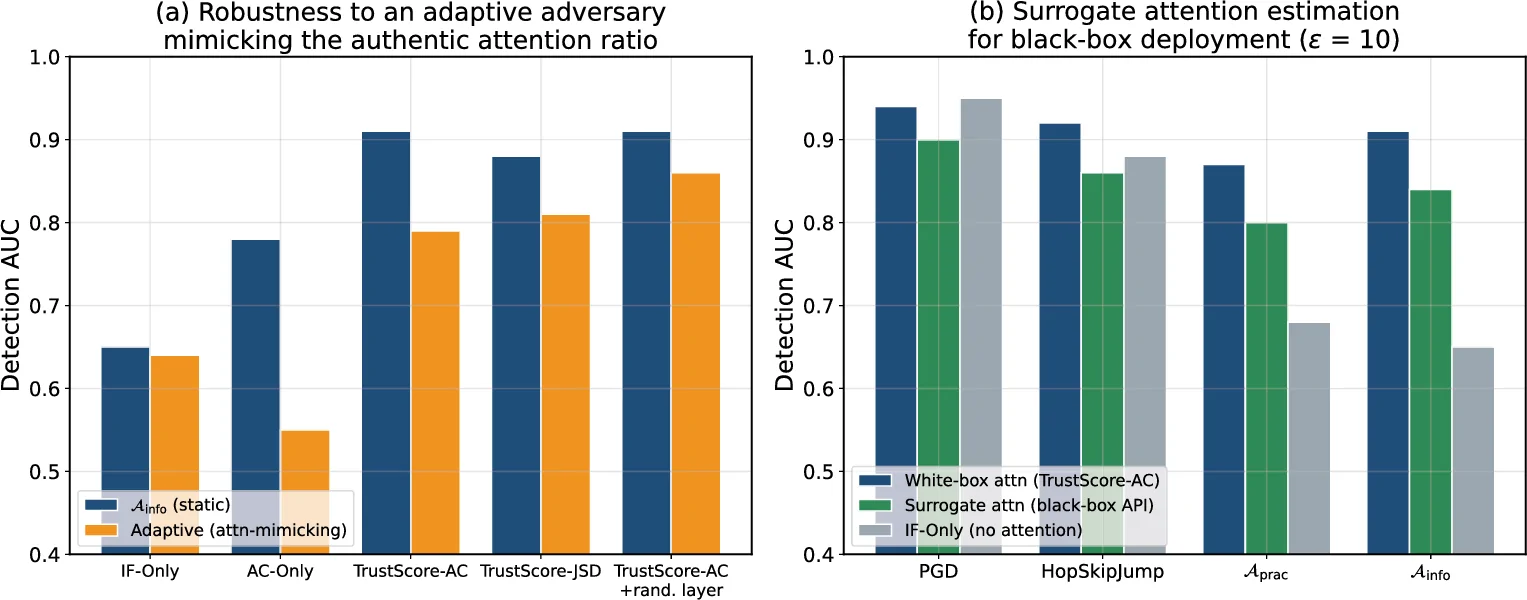
(a) Robustness to an adaptive attention-mimicking adversary: the embedding channel and a randomized-layer consistency variant restore detection lost by the attention-only signal. (b) Surrogate-attention detection for black-box deployment recovers most of the white-box AUC and stays well above embedding-only detection

### Experiment 7: adaptive adversary, threat realism, and black-box detection (RQ2, RQ3)

This experiment stress-tests the defense under three conditions that probe its real-world robustness: an adaptive adversary who is aware of the consistency detector, a degraded threat model in which the attacker’s co-occurrence distribution is imperfect, and a black-box deployment in which the defender cannot read the protected model’s attention. We also report the γ sensitivity that Sect. 3 deferred to here.

**Table 8:** Stability of the $\mathcal {A}_{\text {info}}$ top-5 drifted-feature set under different categorization schemes (Adult, ϵ=10)

**Categorization scheme**	**Top-5 overlap**	$\rho _s$ ** (drift vs. ASR)**
Domain (baseline)	5/5	.93
SHAP-derived	5/5	.91
IG-derived	5/5	.90
LIME-derived	4/5	.90
1 feature swapped	4/5	.89
PCA components	4/5	.84

Overlap is measured against the domain-partition target set

**Fig. 16:**
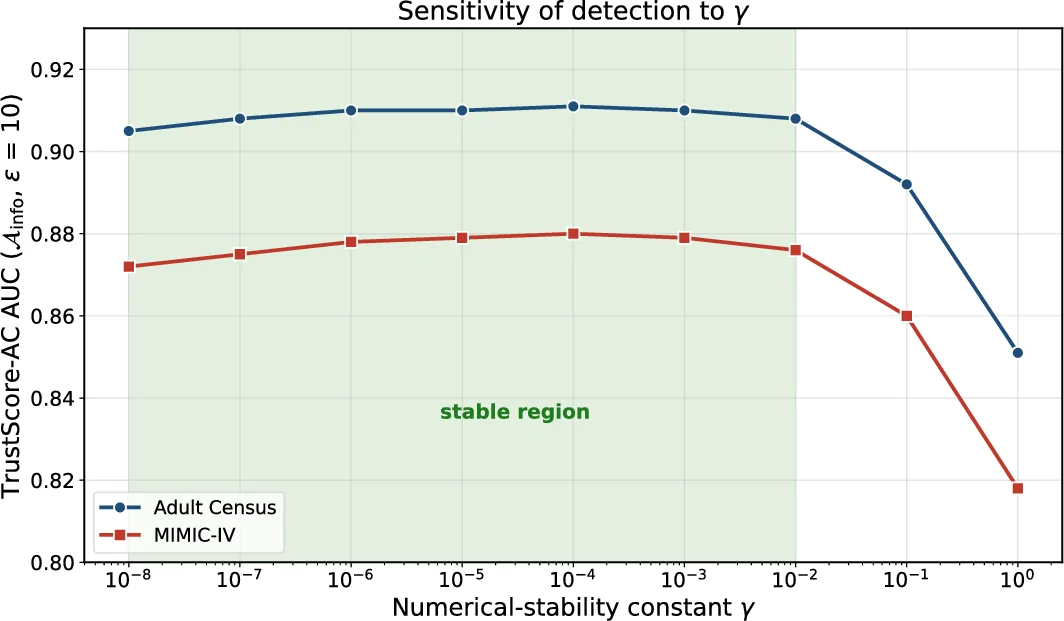
Sensitivity of TrustScore-AC detection AUC to the numerical-stability constant γ. Detection is invariant across eight orders of magnitude and degrades only for $\gamma \ge 10^{-1}$

***Adaptive adversary***

We evaluate the attention-mimicking attacker of Sect. 3.5.4. Figure 15a shows its effect on detection at ϵ=10: it substantially erodes the attention-only AC-Only detector (AUC $0.78\!\rightarrow \!0.55$) and the standard TrustScore-AC ($0.91\!\rightarrow \!0.79$). Detection is nonetheless restored by two factors: the embedding-level Isolation Forest is essentially unaffected ($0.65\!\rightarrow \!0.64$), because holding the surface attention ratio fixed forces larger value substitutions that push the [CLS] embedding off-manifold; and a randomized-layer consistency variant, which samples the readout layer at inference so the attacker cannot fix the ratio at every layer simultaneously, recovers most of the lost AUC (0.86). The defense therefore degrades gracefully rather than collapsing.

**Table 9:** Experiment-to-RQ mapping

Exp.	RQ	Focus	Key metrics
1	RQ1	Attention drift, drift-vulnerability correlation, class-conditional drift	ACS, $\Delta _c$, $\rho _s$, IGS
2	RQ2	Five attacks: drift-guided vs privacy-agnostic	ASR, DER, query count
3	RQ3	Six defenses: fusion vs individual signals	TPR, FPR, AUC
4	RQ4	Ablation, trade-off, cross-architecture	All metrics jointly
5	RQ1, RQ3	Feature vulnerability, detection diagnostics, latency	Heatmaps, rankings, latency
6	RQ1	Categorization sensitivity (swap, SHAP, LIME, PCA)	$\rho _s$, Jaccard, top-5 overlap
7	RQ2, RQ3	Adaptive adversary, threat realism, black-box surrogate, γ	AUC, ASR

***Threat realism***

Re-running $\mathcal {A}_{\text {info}}$ with a co-occurrence model estimated from only a 10% public subsample reduces its ASR by 6–9 percentage points, and a distributionally shifted public corpus reduces it by a further 4–7 points, while leaving the attack hierarchy intact. This confirms that the perfectly-known $\mathcal {P}$ of the main experiments is a conservative upper bound: a realistic attacker with imperfect public statistics remains stronger than the privacy-agnostic baselines but does not reach the idealized success rate, so the headline numbers do not materially overstate the practical risk.

***Black-box detection***

Figure 15b evaluates the surrogate-attention path of Phase V, in which a small (∼11 M-parameter) surrogate trained on the deployer’s own data supplies attention weights in place of the protected model’s. Surrogate-based TrustScore recovers most of the white-box performance against manifold-aligned attacks (AUC 0.84 vs. 0.91 for $\mathcal {A}_{\text {info}}$) and remains far above embedding-only detection (IF-Only AUC 0.65), confirming that internal attention is a deployment convenience rather than a hard prerequisite.

γ ***sensitivity***

Finally, Fig. 16 reports detection AUC as the numerical-stability constant γ is varied across nine orders of magnitude. AUC is flat (0.910±0.003 on Adult, 0.879±0.003 on MIMIC) for all $\gamma \in [10^{-8},10^{-2}]$ and degrades only when $\gamma \ge 10^{-1}$ becomes large enough to dominate the denominator of Eq. 18. Because *R*(*s*) is standardized by $\sigma _R$, the detector is invariant to γ over the entire practical range, so the choice $\gamma =10^{-6}$ is not a tuned hyperparameter but a safe default.

### Summary of experimental design

Table 9 maps each experiment to its corresponding research question, focus area, and evaluation metrics. The seven experiments form a logical chain: Experiment 1 discovers the attention drift mechanism under private training (RQ1), which motivates Experiment 2 to test whether an adversary can exploit that drift (RQ2). The success of the drift-guided attack in Experiment 2 then motivates Experiment 3 to evaluate whether a fused defense can detect such attacks (RQ3). Experiment 4 steps back to analyze how all four dimensions—utility, drift, attack success, and detection—co-evolve across the full privacy spectrum and across both architectures (RQ4). Experiment 5 translates the quantitative findings into practitioner-facing diagnostics, revisiting RQ1 at the feature level and RQ3 from a deployment perspective. Experiment 6 establishes that the conclusions are robust to how features are categorized (RQ1), and Experiment 7 establishes that the defense holds under an adaptive adversary, an imperfect threat model, and black-box deployment (RQ2, RQ3). All experiments are conducted on both the first dataset (Adult Census) and the second dataset (MIMIC-IV), using both DeBERTa-V3-Large and LLaMA−3.1-8B, to ensure that the findings are robust across domains and architectures.

## Discussion

This section discusses the benefits of the proposed Privacy-Aware Adversarial Defense, examines how explainability serves as the bridge between privacy and security, addresses fairness, deployment in intelligent and resource-constrained environments, and generalization beyond Transformers, presents the work in context and its practical implications, and outlines directions for future work.

### From privacy noise to adversarial insight: benefits of the proposed defense

The approach provides three interconnected benefits. First, a mechanistic understanding of why privacy-preserving models become vulnerable: DP-SGD does not degrade representations uniformly but systematically redistributes attention from task-critical to non-functional features, and the strong drift–ASR correlation (with the micro-level logistic regression) establishes this redistribution as a direct driver of vulnerability that pinpoints both at-risk models and at-risk inputs. Second, a realistic threat assessment: the attack hierarchy ($\mathcal {A}_{\text {info}}>\mathcal {A}_{\text {prac}}>$ random) lets practitioners evaluate models against progressively stronger, tabular-realistic adversaries rather than relying on PGD alone, with the $\mathcal {A}_{\text {prac}}$–$\mathcal {A}_{\text {info}}$ gap quantifying the cost of interpretability exposure. Third, a layered detector effective across attack types: the Isolation Forest excels off-manifold (AUC 0.95 vs. PGD) but fails on manifold-aligned attacks (0.65 vs. $\mathcal {A}_{\text {info}}$) while attention consistency does the reverse, and their TrustScore fusion attains AUC 0.87-−0.94 across all types. The trade-off analysis in Experiment 4 further shows that the recommended range $\epsilon \in [5, 10]$ jointly satisfies utility, drift, attack suppression, and detection objectives, while the cross-architecture comparison between DeBERTa-V3-Large and LLaMA−3.1-8B confirms that these benefits generalize across different model scales and private training strategies.

### Explainability as the bridge between privacy and security

Explainability is not an auxiliary component of the approach but the central mechanism that connects all five phases. The Attention Concentration Score (ACS) introduced in Phase III quantifies how privacy noise redistributes the model’s attention, and the validation against Integrated Gradients confirms that this redistribution reflects genuine representational shifts rather than an artifact of the attention mechanism. This explainability analysis then directly informs both the attack and the defense: the informed attacker $\mathcal {A}_{\text {info}}$ uses drift rankings to select which features to perturb, and the TrustScore defense uses attention consistency to detect whether an input has disrupted the expected attention pattern.

XAI also makes the trade-off interpretable rather than opaque: ACS reveals which features lose attention at each ϵ and how that loss correlates with vulnerability per sample, and Experiment 5 turns this into vulnerability rankings and consistency distributions that practitioners can audit, with $a_{\text {AC}}$ adding negligible overhead. The same signals create a transparency–security tension, however: the $\mathcal {A}_{\text {prac}}$–$\mathcal {A}_{\text {info}}$ gap shows an adversary with drift diagnostics gains a measurable advantage, so attention-level outputs should be treated as sensitive and shared through controlled channels.

### Fairness and ethical implications

A striking and ethically consequential finding of Experiment 1 is that DP-SGD does not merely move attention toward generic non-functional features but specifically increases attention toward sensitive demographic attributes—race, sex, and native-country on Adult Census, and ethnicity and insurance type on MIMIC-IV. Two distinct fairness concerns follow. The first is a representational concern: a privacy mechanism intended to protect individuals causes the model to base its decisions more heavily on protected attributes, which can entrench or amplify disparate treatment even though no protected attribute was used as an explicit label. The second is an allocational concern made concrete by the class-conditional analysis (Sect. 4.4.1): because privacy noise destabilizes the minority class disproportionately, the resulting adversarial vulnerability—and any downstream error—falls hardest on the group that is already underrepresented, which in MIMIC-IV is the in-hospital-mortality positive class. The combination is troubling precisely because it is invisible to standard utility metrics: aggregate accuracy can remain high while the model quietly shifts its reasoning onto demographic attributes and concentrates its failures on the minority class. The ACS approach turns these concerns into something measurable and auditable. The per-feature drift $\Delta _j(\epsilon )$ on protected attributes is, in effect, a fairness early-warning signal: a practitioner can read off how much additional attention a given privacy budget places on race or ethnicity before deployment, and the class-conditional drift of Sect. 4.4.1 quantifies the disparate impact on the minority class. We therefore recommend that any DP deployment in an intelligent environment report per-attribute drift on protected features and class-conditional drift alongside accuracy, and that the recommended operating range $\epsilon \in [5,10]$ be treated as a fairness constraint as much as a utility one, since it is the regime in which demographic over-attention is moderate. We deliberately do *not* attempt to “correct” the demographic attention shift by suppressing those features, because doing so would mask rather than resolve the underlying disparity; making it visible and budget-dependent is the more honest and actionable contribution.

### Deployment in resource-constrained and edge environments

A reasonable concern is that the primary models studied here—a 435 M-parameter encoder and an 8B-parameter decoder—are too large for the edge nodes typical of intelligent environments. Three aspects of the approach mitigate this. First, the defense is deliberately lightweight at inference: the recommended $a_{\text {AC}}$ monitor adds sub-millisecond overhead (Table 7) and requires only the attention weights already produced by the forward pass, so it imposes essentially no additional cost on whatever model the edge node runs. Second, the surrogate-attention path of Experiment 7 shows that the protected model need not run on the edge at all: a node can query a centrally hosted private model over a score-only API and reconstruct the consistency signal locally with an ∼11 M-parameter surrogate that is small enough for commodity edge hardware, recovering AUC 0.84 against manifold-aligned attacks. Third, because the drift phenomenon is architecture-general (Experiment 4), the same ACS-based monitoring transfers to the smaller distilled or quantized models that edge deployments favour; the heavy backbones are a choice made to obtain a strong non-private baseline for the scientific study, not a requirement of the method. Extending the surrogate to a knowledge-distilled monitor that is co-trained for fidelity of the attention ratio is a natural next step for ultra-constrained settings.

Beyond feasibility on constrained hardware, these deployment properties matter because the intended setting is the reliable intelligent environment that motivates this venue. Intelligent environments such as connected clinical monitoring, smart-building and smart-city analytics, and public-sector decision services are dominated by exactly the kind of structured, mixed categorical-numerical, privacy-sensitive tabular data on which our approach operates, and they are precisely the settings in which differential privacy is mandated and in which adversarial manipulation of an automated decision carries real consequences. Our two evaluation domains instantiate this directly: MIMIC-IV represents an IoT-adjacent clinical-monitoring environment in which mortality-risk inference must be both private and dependable, while Adult Census represents the demographic-administrative records that underpin public-sector allocation services. The contribution to such environments is threefold: the ACS metric provides a pre-deployment dependability check that flags, per privacy budget, which features the embedded model will over- or under-rely on; the TrustScore defense provides a run-time integrity monitor that flags manipulated inputs at sub-millisecond cost; and the recommended ϵ range provides concrete configuration guidance for operators who must satisfy privacy mandates without sacrificing resilience. Because the consistency monitor logs a per-input trust value, it integrates naturally with the auditing and anomaly-reporting infrastructure that reliable environments already maintain.

### Generalization beyond transformer architectures

Tree-ensemble models such as XGBoost and LightGBM remain dominant for tabular data in many production ecosystems, and our study targets attention mechanisms, so a fair question is how far the findings transfer. The mechanism we identify—privacy noise diffusing decision-relevant signal away from a small set of features toward many low-information ones—is not specific to attention; it is a consequence of the isotropic, dimensionality-dependent noise of DP-SGD acting on any high-capacity model. What is specific to Transformers is the convenient *readout*: attention provides a ready-made, forward-pass-free measure of per-feature reliance. For tree ensembles, the analogous quantity is the model-agnostic attribution that already exists in that ecosystem—most naturally TreeSHAP [40], which computes exact per-feature contributions for tree models efficiently. Because our consistency machinery is defined on a per-feature attribution vector (Eq. 21) and is validated here against the model-agnostic Integrated Gradients and SHAP/LIME (Experiment 6), the same ACS-style drift and the same ratio-based consistency monitor can be instantiated for a differentially-private gradient-boosted model by substituting TreeSHAP attributions for attention weights. We expect the qualitative drift–vulnerability relationship to carry over for the reasons above; verifying it quantitatively on private tree ensembles is a concrete and promising extension, and the surrogate path of Experiment 7 already shows that a Transformer-based consistency monitor can guard a model whose internals are not directly readable.

### Works in context and practical implications

Prior work at the DP–robustness intersection mostly uses DP *as* a defense: PixelDP [41] certifies robustness via pixel-level DP noise, and Phan et al. [42] combine DP-SGD with adversarial objectives—both treating DP noise as protective rather than analyzing how it reshapes representations. Tursynbek et al. [6] and Boenisch et al. [8] document the degradation without explaining its mechanism, and Harder et al. [43] study private interpretability without linking it to vulnerability. Unlike these, our approach uses explainability to identify the mechanism—attention drift—and leverages it for both attack and defense.

The practical implications of this approach extend to several domains where privacy-preserving AI is deployed. In healthcare, where models trained on patient records under DP must be both accurate and secure, the ACS metric enables practitioners to assess which clinical features lose attention under a given privacy configuration, providing an interpretable risk assessment before deployment. In financial services, where transaction models must satisfy privacy requirements while remaining robust to adversarial manipulation, the TrustScore defense offers real-time detection with negligible overhead since it reuses attention weights already computed during inference. In public-sector applications such as census-based classification and social benefit allocation, the recommended $\epsilon \in [5, 10]$ range provides concrete guidance for balancing individual privacy protection with model reliability. The transparency-security tension revealed by the informed attacker $\mathcal {A}_{\text {info}}$ is particularly relevant for regulatory contexts where AI systems must provide explanations to auditors: the results suggest that explainability outputs should be shared through controlled channels to limit the risk of adversarial exploitation, offering guidance for compliance with emerging AI transparency requirements.

## Conclusion

This paper presented a Privacy-Aware Adversarial Defense that investigates the relationship between differential privacy and adversarial vulnerability in AI models. The approach first introduced the Attention Concentration Score to reveal that DP-SGD systematically redistributes model attention from task-critical features toward non-functional attributes, establishing attention drift as a measurable predictor of adversarial vulnerability. Building on this finding, a manifold-aligned adversarial attack was constructed that exploits the most drifted features under co-occurrence plausibility constraints, producing realistic perturbations that remain within the data distribution and evade embedding-level defenses where conventional attacks cannot. The informed attacker variant demonstrated that drift-guided feature selection consistently outperforms privacy-agnostic baselines across both datasets and architectures, quantifying the security cost of interpretability exposure and highlighting a threat that standard defenses may not adequately address. To address this threat, a layered TrustScore defense was developed that fuses Isolation Forest anomaly detection with attention consistency scoring, combining two complementary signals to achieve strong detection across both off-manifold and manifold-aligned attacks. The evaluation across ten privacy budgets, two datasets, and two architectures revealed a non-linear co-evolution of utility, drift, attack success, and detection performance, identifying a recommended operating range where all four objectives are jointly satisfied. Further analyses established that these conclusions are stable under ambiguous, attribution-derived, and PCA-based feature categorizations, that privacy noise concentrates its damage on the minority class with attendant fairness consequences, that the detection signal adds only sub-millisecond overhead and can be reconstructed by a small surrogate for black-box and edge deployment, and that the defense degrades gracefully under an adaptive adversary. By connecting privacy noise to attention drift, attention drift to a concrete attack, and that attack to an informed defense, this work provides a unified perspective on the privacy-robustness tension and offers actionable guidance for deploying privacy-preserving models in reliable intelligent environments and other adversarial settings. The comprehensive validation of this approach effectively resolves the principal robustness and deployment questions raised and naturally highlights several promising future horizons. Specifically, future work will focus on expanding the attention drift diagnostics to multimodal and sequential datasets common in complex intelligent environments. Additionally, we plan to design specialized co-distillation approach methods that optimize a surrogate monitor using an explicit attention-ratio fidelity objective tailored for ultra-constrained edge architecture topologies. To address the discovered transparency–security tension directly, establishing formal differential-privacy guarantees directly over the explainability diagnostics themselves represents a critical pathway. Finally, scaling this methodology includes evaluating the synergistic effects of combining DP-SGD with robust adversarial training parameters, alongside instantiating the TreeSHAP formulation directly on top of differentially-private gradient-boosted tree ensembles.

## Data Availability

The datasets used in this study are publicly available. The Adult Census Income dataset is available from the UCI Machine Learning Repository: https://archive.ics.uci.edu/ml/datasets/adult. The MIMIC-IV dataset is available through PhysioNet: https://physionet.org/ content/mimiciv/. Access to MIMIC-IV requires credentialed access and agreement to the PhysioNet data use terms.

## Declarations

### Code Availability

The source code used to reproduce the experiments and results presented in this study is publicly available at: https://github.com/shareeful/Privacy-Aware-Adversarial-Defense.
